# Evaluating completeness, coherence, and consistency of genome-scale function annotations

**DOI:** 10.1093/bib/bbag336

**Published:** 2026-06-29

**Authors:** Rund Tawfiq, Maxat Kulmanov, Robert Hoehndorf

**Affiliations:** Biological and Environmental Sciences & Engineering (BESE) Division, King Abdullah University of Science and Technology, 4700 KAUST, Thuwal 23955, Saudi Arabia; KAUST Center of Excellence for Smart Health (KCSH), King Abdullah University of Science and Technology, 4700 KAUST, Thuwal 23955, Saudi Arabia; KAUST Center of Excellence for Smart Health (KCSH), King Abdullah University of Science and Technology, 4700 KAUST, Thuwal 23955, Saudi Arabia; Computer, Electrical and Mathematical Sciences and Engineering (CEMSE) Division, King Abdullah University of Science and Technology, 4700 KAUST, Thuwal 23955, Saudi Arabia; KAUST Center of Excellence for Generative AI, King Abdullah University of Science and Technology, 4700 KAUST, Thuwal 23955, Saudi Arabia; Biological and Environmental Sciences & Engineering (BESE) Division, King Abdullah University of Science and Technology, 4700 KAUST, Thuwal 23955, Saudi Arabia; KAUST Center of Excellence for Smart Health (KCSH), King Abdullah University of Science and Technology, 4700 KAUST, Thuwal 23955, Saudi Arabia; Computer, Electrical and Mathematical Sciences and Engineering (CEMSE) Division, King Abdullah University of Science and Technology, 4700 KAUST, Thuwal 23955, Saudi Arabia; KAUST Center of Excellence for Generative AI, King Abdullah University of Science and Technology, 4700 KAUST, Thuwal 23955, Saudi Arabia

**Keywords:** systems biology, functional genomics, protein function, Gene Ontology

## Abstract

Protein function annotation traditionally follows a reductionist approach, assigning functions to individual proteins acting in isolation. This treats each annotation as an independent fact, disconnected from the broader biological system. However, proteins operate within integrated networks where their functions depend on genomic context and interacting partners. This needs to be reflected in function annotation and evaluation frameworks. We assess whether annotated protein functions could plausibly coexist within a living organism. To achieve this goal, we formalize three criteria grounded in systems biology principles: completeness (presence of essential functions), coherence (satisfaction of functional dependencies), and consistency (absence of mutually exclusive functions). We applied this framework to manually curated function annotations from six model organisms and computational function predictions from seven methods. While model organism annotations largely satisfied our constraints, computational function prediction methods systematically failed to produce biologically plausible genome-scale annotations. Our review reveals a measurable gap between the per-protein objectives of current annotation methods and the system-level criteria that an annotation set must satisfy to describe a viable organism. Our evaluation framework grounded in systems biology principles provides quantitative metrics for evaluating biological plausibility and establishes a foundation for developing system-aware annotation approaches. Augmenting protein-level annotation with system-level criteria offers a tractable path to improving annotation of the rapidly growing collection of sequenced genomes and metagenomes.

## Introduction

The Gene Ontology (GO) [1] has been adopted to describe and compare functions across species, and to capture the diversity of biological functions across life. As one of the main methods to describe biological functions, the GO has been the foundation of comparative and functional genomics and other functional studies in biology. The main use of GO is to assert that proteins and other gene products have certain functions. These assertions are usually created by highly trained curators who read literature, extract functional information that can be coded with GO, and create manual assertions [2].

Experimental characterization of protein functions does not scale with the amount of protein sequences becoming available, as experimental validation of protein function remains a low throughput process. Therefore, computational methods for function prediction have been developed following many different approaches [3]. These approaches can be categorized by the biological principles they aim to exploit. Methods based on sequence homology, such as BLAST2GO [4] or eggNOG-mapper [5], compare novel protein sequences to databases of well-characterized proteins and assign function based on sequence similarity; methods such as InterProScan [6] match protein sequences against established collections of domain, family, and motif signatures; and machine learning approaches use multiple types of protein features and learning paradigms to predict their functions [7].

The performance of these methods is evaluated in competitions such as the Critical Assessment of Functional Annotation (CAFA) [8]. CAFA provides a standardized framework for assessing protein function prediction based on predefined benchmarks [9]. While CAFA has advanced the field by providing standardized benchmarks, its evaluation paradigm focuses exclusively on the accuracy of individual protein predictions, without considering whether the collective set of predictions for an organism makes biological sense.

Current annotation models, prediction tools, and evaluation frameworks all share the same fundamental paradigm: they treat proteins as individual, isolated entities instead of parts of integrated biological systems. The GO annotation model, protein function prediction tools, and function prediction evaluation methods all focus on assigning functions to individual proteins, where the protein is usually given by its amino acid sequence.

However, the majority of functions in the GO cannot reasonably be predicted from a single protein sequence. Specifically, the GO has three sub-ontologies: molecular functions, biological processes, and cellular components [1]. Generally, molecular functions, which include functions such as binding to specific compounds or enzymatic activities, can be determined for a single protein. Although some biological processes may be mediated by a single protein (e.g. single-step metabolic reactions), most biological processes require more than one protein to participate; examples are processes corresponding to biological pathways where different proteins are required to perform different steps of the pathway. The presence of a single protein *p* in an organism *O* that is identical to another $p^{\prime}$ in another organism $O^{\prime}$ may provide a degree of evidence for the occurrence of process *P* in *O*, but this evidence is often insufficient for a definitive prediction. This is because many biological processes *P* require other proteins, and the identification of *p* does not provide information regarding the presence of the other necessary components.

An exception to this protein-centric paradigm is the development of GO Causal Activity Models (GO-CAMs) [10], which explicitly represent how multiple proteins coordinate to realize biological processes. However, GO-CAMs remain limited in scope—they primarily focus on human biology, require manual curation, and only a small number currently exist.

Systems biology approaches attempt to shift toward proteome-level annotation through quantitative simulations such as Flux Balance Analysis, but these often rely on mass balance and arbitrary flux bounds in the absence of experimental data [11]. Methods like GEM-PRO replace these bounds with biophysical limits (e.g. enzyme abundance), but remain data-intensive and largely species-specific [12].

With the increased availability of genomes and metagenomes, we now have the opportunity to reformulate the task of protein function annotation, prediction, and evaluation and move toward proteome-scale protein function characterization. Given a set of proteins (the “proteome”), the task is to describe, predict, and evaluate the functions of all proteins of the set. This reformulation can allow us to ask new questions: Does the set of predicted functions contain all essential functions for life? Are functional dependencies satisfied? Are there combinations of functions that would be impossible in living systems?

Proteome- or genome-scale perspective become important when we consider that organisms are not random collections of proteins, but evolved systems with specific functional requirements and constraints. While the reductionist paradigm used in databases like UniProt [13] and other GO annotations [14] has been useful to describe our molecular understanding, reductionism does not allow us to capture the emergent properties that arise from protein interactions and system-level organization, and may lead to annotations that do not respect system-level properties. Previous efforts have attempted to bridge this gap at the protein level by calculating functional similarity to ensure consistency of individual annotations [15]. However, this is focused on the validity at the protein level, and does not utilize logical axioms of the GO to verify whether the collective set of functions satisfies the requirements for a viable system.

We define three fundamental properties that functions for a set of proteins within a proteome should have in order to allow these functions to coexist in a biological organism. Our approach relies on constraints already encoded in the GO structure, combined with universal biological principles that apply across all cellular life. First, “completeness” ensures the presence of functions essential for life: every viable bacterial genome, for instance, must encode proteins that together perform processes such as DNA replication, transcription, and translation. Second, “coherence” guarantees that functional dependencies are satisfied—if function *A* requires function *B*, both must be present within the proteome, e.g. predicting that a genome encodes a late-stage step of *nitrogen fixation* (GO:0009399) requires that the same genome also encodes proteins for the preceding steps of the pathway. Third, “consistency” prevents the co-occurrence of mutually exclusive functions: an annotation set should not simultaneously assign *photosynthesis* and *neuron development* to proteins of the same genome, as these correspond to functions restricted to disjoint groups of organisms. Our framework specifically addresses bacteria, archaea, and eukaryotes, where these constraints reflect fundamental requirements for autonomous life [16]; the operational form of each measure is presented in the next section, with the underlying notation and the formal logical definitions consolidated in its final subsection (Logical formulation).

We design an evaluation framework to evaluate function annotations at the genome-scale, where we first formalize these three properties as logical constraints that are based on the GO, thereby directly relating our notion of proteome-level “function” to existing protein-level annotations based on the GO. We then apply our framework to assess and review bacterial genome annotations. We test whether gold standard, curated annotations of model organisms satisfy the system-level constraints in our framework, and we evaluate different protein function prediction methods according to our constraints.

This application of our evaluation framework reveals a substantial contrast between manually curated annotations and computational predictions. Manually curated model organism annotations largely satisfy system-level constraints, with violations primarily from pathogen proteins annotated with host-specific functions (e.g. wound healing). In contrast, current function prediction methods systematically fail to satisfy these constraints across all three criteria—no method produces annotations that would represent a biologically viable organism; while function prediction methods may accurately predict individual protein functions, they fail to capture the system-level requirements of living organisms.

Our evaluation measures and evaluation results are implemented as Free Software available at https://github.com/bio-ontology-research-group/GAEF. We anticipate this review and evaluation framework will guide the development of next-generation function annotation methods that explicitly consider genome-scale constraints.

## Evaluation framework

This section presents the three measures of our evaluation framework. Each measure is described in operational form: the biological intuition behind the measure, an illustrative example, and the computational metric we use to score genome-scale annotations. The notation underlying these measures, together with their formal logical definitions and ontological grounding, are consolidated in Logical formulation (*Logical formulation*) at the end of this section. Readers interested only in the operational use of the measures may proceed without that subsection without loss of understanding; readers interested in the formal grounding will find all of the relevant predicates, the *hF*/*in* relations, and the formal definitions of the three measures collected there.

### Completeness

Our first measure is “completeness,” which captures the idea that certain functions are necessary for life, and that one or more proteins involved in such essential functions must therefore be present in every viable genome. A genome’s annotations are complete, relative to a set $\mathcal{R}$ of functions deemed essential for viability, if for every $F \in \mathcal{R}$ at least one protein in the genome is annotated with *F* (or one of its more specific sub-classes). The corresponding formal definition is given in Logical formulation (Definition 1).

While there are certain essential functions that are shared by all life (reproduction, energy metabolism, DNA replication, etc.), some functions are only shared by certain types of organisms (e.g. C4 photosynthesis only in plants, or lactation only in mammals). The definition can be adapted to incorporate taxon-level essential functions; for instance, mycolic acid biosynthesis is essential for cell wall integrity, and thus viability, of *Mycobacterium* [17].

Practical application of the completeness criterion requires identifying which functions should be considered essential in a bacterial genome. To identify essential GO classes, we manually mapped functional categories from the *Mycoplasma mycoides* genome in Syn1.0 [18] to their corresponding GO classes (Table 1). The Syn1.0 genome, created through systematic transposon mutagenesis experiments, represents a minimal bacterial genome required for viability under laboratory conditions. It is useful for defining essential functions as it represents an empirically validated minimal gene set for bacterial organisms. We grouped the resulting GO classes into five broad categories: Core, Defense, Glucose Metabolism, Nutrient Uptake, and Environmental Adaptation. The “Core” category includes functions that are universally essential in bacteria, while the remaining categories represent functions that vary depending on metabolic strategies, environmental conditions, and stress responses. The curated set of “Core” essential functions formed the basis for the target GO classes that we used to quantify the presence of essential functions in bacterial genomic assemblies.

**Table 1:** Functional categories from syn1.0 manually mapped to the most relevant GO class.

Category	Function	GO class name	GO class
Core	DNA metabolism	DNA metabolic process	GO:0006259
	DNA replication	DNA replication	GO:0006260
	DNA repair	DNA repair	GO:0006281
	Transcription	DNA-templated transcription	GO:0006351
	Translation	Translation	GO:0006412
	Cell division	Cell division	GO:0051301
	Chromosome segregation	Chromosome segregation	GO:0007059
	Ribosome biogenesis	Ribosome biogenesis	GO:0042254
	Protein folding	Protein folding	GO:0006457
	Protein export	Protein transport	GO:0015031
	RNA metabolism	RNA metabolic process	GO:0016070
	rRNA modification	rRNA modification	GO:0000154
	tRNA modification	tRNA modification	GO:0006400
	RNA (rRNAs, tRNAs, small RNAs)	RNA biosynthetic process	GO:0032774
	Proteolysis	Proteolysis	GO:0006508
	Metabolic processes	Metabolic process	GO:0008152
	Membrane transport	Transmembrane transport	GO:0055085
	Lipid salvage and biogenesis	Lipid metabolic process	GO:0006629
	Transport of nonglucose carbon sources	Organic acid transport	GO:0015849
	Catabolism of nonglucose carbon sources	Organic acid catabolic process	GO:0016054
	Redox homeostasis	Cell redox homeostasis	GO:0045454
	Regulation	Regulation of biological process	GO:0050789
Glucose metabolism	Glycolysis	Glycolytic process	GO:0006096
	Glucose transport	Glucose transmembrane transport	GO:1904659
Environmental adaptation	Mobile elements	Transposition	GO:0032196
	DNA topology	DNA conformation change	GO:0071103
	Lipoprotein	Lipoprotein metabolic process	GO:0042157
Nutrient uptake	Cofactor transport and salvage	Vitamin transmembrane transporter activity	GO:0090482
	Acylglycerol breakdown	Acylglycerol catabolic process	GO:0046464
	Nucleotide salvage	Nucleotide salvage	GO:0043173
Defense	DNA restriction	DNA restriction-modification system	GO:0009307
	Efflux	Export across plasma membrane	GO:0140115

To quantify the presence of these essential GO classes in function prediction methods, we annotated each genome in our benchmark set with GO classes predicted by each method and expanded these annotations by including the ancestor classes (using “is a,” “part of,” and “occurs in”) for each protein according to the GO hierarchy. For each GO class *F* we marked as essential, we computed the percentage of genomes *g* where at least one protein with function *F* is present, thereby quantifying the degree to which each genome satisfies our completeness criterion.

### Coherence

Other constraints on genome-scale function annotation rely on interactions between functions, i.e. where the prediction of one function necessitates the presence of another. These dependencies can be on the level of a single protein where predicting a function for a protein necessitates that the protein also has another function. For example, if a protein is localized to a specific part of the cell such as the *nucleolus* (GO:0005730), it must also be localized to the *nucleus* (GO:0005634). The dependencies can also be on the level of a genome where predicting a function for one protein in the genome necessitates that there is another protein in the same genome with the dependent function. For instance, predicting that a genome encodes an enzyme for a late-stage step in *nitrogen fixation* (GO:0009399) requires that the genome also contains proteins annotated with the preceding steps in the pathway. We call this property *coherence*.

More generally, given a set $\mathcal{C}$ of pairs of dependent functions, a genome’s annotations are coherent with respect to $\mathcal{C}$ if, for every pair $(F_{1}, F_{2}) \in \mathcal{C}$, the presence of $F_{1}$ in the genome implies the presence of $F_{2}$. Coherence has both a protein-level form (in which both functions must be carried by the same protein) and a genome-level form (in which the dependent function must be present anywhere in the proteome); protein-level coherence implies genome-level coherence but not the other way around. The corresponding formal definition is given in Logical formulation (Definition 2). One source of the set $\mathcal{C}$ are the axioms of GO itself, which yield dependent functions for both forms; the GO “true path rule” [14] already enforces protein-level coherence by propagating annotations across the GO hierarchy, so in what follows we focus on genome-level coherence.

#### Process and pathway coherence

The intuition of process and pathway coherence is that there are processes that have necessary parts (or “steps”) that must be executed for the entire process to take place; however, these steps can be performed by (potentially) multiple proteins, not necessarily by a single one. This results in a genome-level coherence constraint. An example of this constraint is *Lipopolysaccharide immune receptor activity* (GO:0001875), which requires, through a “has part” (RO:0000051) relation, *Lipopolysaccharide binding* (GO:0001530).

We can use the axioms in the GO to identify such genome-scale dependencies using axioms involving the “has part” (RO:0000051) relation. We define the set of dependent functions $\mathcal{C}_{hp}$, for every GO class *F*:


\begin{align*} & \mathcal{C}_{hp} = \{ (C,F) | C \sqsubseteq \exists{^{\prime}\text{has part}^{\prime}}.F\} \end{align*}

We used the ELK reasoner [19] to find all sub-classes of the class “has part some X” (for every class X in GO), retaining only direct sub-classes; use of ELK is necessary as we are not only interested in asserted dependencies but also those that can be inferred deductively. As a result, for the GO version we processed, we obtained 5038 pairs of classes connected through “has part” relations. To assess process coherence with respect to these pairs of classes, we calculated, at the genome level, the proportion of “has part” relations that were satisfied in a genome’s annotations.

Let $\mathcal{R}$ be the set of all $(C,F) \in \mathcal{C}_{hp}$ for which *C* is present in genome *g* (i.e. at least one protein in *g* is annotated with class *C*), and let $\mathcal{M} \subseteq \mathcal{R}$ be the subset of pairs in which the required part *F* is missing (i.e. no protein in *g* is annotated with class *F*). We then calculated process coherence as the percentage of satisfied dependencies:


\begin{align*}& \mathrm{Coherence}(\mathcal{C}_{hp}, g) = \left(1 - \frac{|\mathcal{M}|}{|\mathcal{R}|} \right) \times 100 \end{align*}

This kind of dependency can be extended to molecular pathways, which represent coordinated series of biochemical reactions [20]. In metabolic pathways, these reactions transform substrates into products through enzyme-catalyzed steps, where each enzyme corresponds to a specific molecular function. Specifically, if a GO class $F_{P}$ is mapped to a pathway $\mathcal{P}$ and some protein *p* in genome *g* is predicted to have $F_{P}$ as function, we assess whether the genome contains proteins with the necessary functions to execute the entire pathway.

Metabolic pathways can be represented as directed graphs where nodes are metabolites and edges are reactions catalyzed by specific enzymes (or other functional proteins). Many pathways contain alternative routes and redundancies that can achieve the same overall transformation through different intermediate steps. This representation can be generalized to signaling pathways, where nodes may represent proteins or protein states (e.g. phosphorylated forms), and edges represent processes such as protein modifications, protein–protein interactions, or regulatory relationships. In both metabolic and signaling contexts, pathways often contain alternative mechanisms (or routes) to achieve the same functional outcome.

We extracted MetaCyc [21] pathway annotations and their corresponding GO classes from the annotation property assertions of GO, resulting in 474 pathways. Using the Pathway Tools API (v28.0) [22], we retrieved each pathway’s structure, reactions, and sub-pathways. We recursively expanded reactions to include sub-pathways, allowing us to build a complete directed graph representing the pathway, where nodes are reactions and edges represent dependencies between steps (as determined by the *get-predecessors* function in Pathway Tools). We mapped each reaction to its EC number using the ec_number attribute from the corresponding PFrame object. For reactions with multiple EC numbers, we generated all possible combinations of EC number paths through the pathway to account for alternative enzymatic routes. We mapped EC numbers to GO classes using http://www.geneontology.org/external2go/ec2go (r16-03-2025), resulting in 1097 successful mappings and 255 unmapped entries.

For each pathway $\mathcal{P}$, we define a “path” as a chain of reactions connecting input metabolites to output metabolites, where each reaction is catalyzed by a specific function (enzyme, transporter, or other protein). We identified all such paths using depth first search on the reaction graph. A path *i* is defined as a sequence of functions $[F_{1}, F_{2}, \dots , F_{k}]$, where each $F_{j}$ corresponds to an edge (reaction) in the graph. We denote the set of all paths in pathway $\mathcal{P}$ as $I_{\mathcal{P}} = {i_{1}, i_{2}, \dots , i_{n}}$. For each path $i \in I_{\mathcal{P}}$, we define $\mathcal{F}_{i}$ as the set of functions required to complete that specific path. A function *F* is considered present in genome *g* if there exists at least one protein *p* in *g* that is annotated with *F*.

We then calculated the completeness of a pathway with respect to a genome by identifying the proportion of required functions in each path that are present in the genome. Because metabolic networks often contain alternative routes that achieve the same biochemical transformation, a pathway is considered coherent if at least one complete path from pathway inputs to outputs exists. Therefore, we score pathway coherence using the maximum completeness across all possible paths:


\begin{align*} \mathrm{Coherence}(\mathcal{P}, g) &= \\& \max_{i \in I_{\mathcal{P}}} \left( \frac{|\{F \in \mathcal{F}_{i}: F \text{ is present in } g\}|}{|\mathcal{F}_{i}|} \right) \end{align*}

This pathway coherence measure implements genome-level coherence by treating each pathway as a set of function dependencies $\mathcal{C}_{\mathcal{P}}$, where functions in the same path are dependent on each other for the pathway’s operation. Instead of a binary determination of coherence, this metric quantifies the degree to which the coherence condition is satisfied for alternative paths through the pathway.

#### Coherence of heteromeric protein complexes

Protein complexes require the presence of multiple interacting proteins. Homomeric complexes can form from multiple copies of the same type of protein, and heteromeric complexes require multiple distinct types of proteins. We evaluated whether heteromeric protein complex annotations are coherent in the sense that, if a protein *p* in genome *g* is annotated with a class representing a heteromeric protein complex, there is at least another protein $p^{\prime}$ ($p^{\prime} \not = p$) in *g* that has the same annotation.

We evaluated the coherence of protein–protein complexes in bacterial genomes using GO annotations. First, we identified protein complex classes (children of *Protein–protein complex*, GO:0032991). We manually reviewed its subclasses and classified them as homomers (complexes of identical protein subunits), heteromers (complexes of distinct protein subunits), or classes that could represent either type. We used this curated set, (available on Github https://github.com/bio-ontology-research-group/GAEF), to identify the type of protein–protein complex.

For each genome, we extracted all proteins annotated with Protein–protein complex (GO:0032991) or any of its child classes. We then classified these annotations into two categories: (1) coherent annotations, which include annotations of homomeric complex classes to any number of proteins, and annotations of heteromeric complex classes to multiple (at least two) different proteins within the same genome; (2) incoherent annotations, which include annotations of heteromeric complex classes to exactly one protein within the genome. For a genome *g* with set of proteins *P*, and for a complex class *C* that is a sub-class of *Protein–protein complex* (GO:0032991), we define the coherence of its annotation as


\begin{align*}& \mathrm{Coherence}(C, g) = \begin{cases} \mathrm{True}, & \mathrm{if} \ C \ \text{is a homomer and at least one}\\ & \text{protein in} \ P \ \text{is annotated with} \ C \\ \mathrm{True}, & \mathrm{if} \ C \ \text{is a heteromer and at least two}\\ & \text{distinct proteins in} \ P \ \text{are annotated with} \ C \\ \mathrm{False}, & \mathrm{otherwise} \end{cases} \end{align*}

For each genome, we calculated protein complex coherence by dividing the number of incoherent complexes (heteromeric complexes annotated to exactly one protein in the genome) by the total number of complex classes annotated in the genome:


\begin{align*}& \mathrm{Incoherent}(g) = |\{C: C \text{ is heteromeric} \land \mathrm{Coherence}(C, g) = \mathrm{False}\}| \end{align*}

\begin{align*}& \mathrm{Total}(g) = |\{C: \text{at least one protein in} \ g \ \text{is annotated with} C\}| \end{align*}

\begin{align*}& \mathrm{ComplexCoherence}(g) = \left(1 - \frac{\mathrm{Incoherent}(g)}{\mathrm{Total}(g)}\right) \times 100 \end{align*}

### Consistency

While coherence captures positive dependencies (required co-occurrence), consistency addresses negative dependencies (mutual exclusion). Coherence captures the notion of dependencies between functions: if there is a protein with function $F_{1}$, there must be a protein with function $F_{2}$. For consistency, we consider another type of dependency where functions exclude each other: having a protein with function $F_{1}$ in a genome excludes having a protein with function $F_{2}$ in the same genome. An example would be the annotation of *photosynthesis* and *neuron development* for proteins on the same genome, or any two functions where one is restricted to multicellular organisms and the other to single-cellular organisms.

Given a set $\mathcal{M}$ of pairs of mutually exclusive functions, a genome’s annotations are consistent with respect to $\mathcal{M}$ if, for every pair $(F_{1}, F_{2}) \in \mathcal{M}$, the presence of $F_{1}$ in the genome rules out the presence of $F_{2}$ in the same genome. As with coherence, consistency has a protein-level form (no single protein carries both functions) and a genome-level form (no two proteins in the same genome carry the two functions). The corresponding formal definition is given in Logical formulation (Definition 3).

To evaluate taxonomic consistency, we use the “in taxon” constraints encoded in GO. These constraints specify which taxa can (“only in taxon,” RO:0002160) or cannot (“never in taxon,” RO:0002161) perform certain functions. We extracted these constraints from the go-computed-taxon-constraints.obo file (r16-03-2025), which provides curated taxonomic restrictions for GO classes [23].

Our approach deliberately avoids comparing predicted functions against the known taxonomy of the sequenced genome. We made this design choice for two reasons. First, when sequencing novel genomes, the precise taxonomic assignment may be uncertain or disputed. Second, in metagenomic applications, sequences may come from unknown or unculturable organisms. By checking for logical consistency among the annotated functions themselves—rather than against a predetermined taxon—our framework remains applicable even when taxonomic identity is unknown.

For each genome, we aggregate all taxonomic constraints across all annotated proteins; for a set of proteins, we obtain the set of positive (only in taxon) and negative (never in taxon) constraints. Given a set of GO function $\mathcal{F}$, let $p_{1},...,p_{n}$ be the set of taxa that occur in positive (only in taxon) restrictions in $\mathcal{F}$, and $n_{1},...,n_{m}$ the set of taxa that occur in negative restrictions. From the taxon constraints, we construct the logical expression $p_{1} \sqcap ... \sqcap p_{n} \sqcap \neg n_{1} \sqcap ... \sqcap n_{m}$ and test whether the resulting class is satisfiable when combined with the axioms of NCBI Taxonomy [24].

Because the ELK reasoner is not able to process negated classes, for each class *C* that occurs in a negative GO taxon constraint, we create a new class $C_{neg}$ that we declare to be disjoint with *C*; the reason for this is that ELK can process disjointness between named classes, but not direct negation. We use these newly created classes to test for satisfiability of the taxon constraints. When a contradiction is detected (i.e. the class expression *C* is unsatisfiable, $C \sqsubseteq \bot$), we utilize ELK’s explanation capability to generate logical justifications for the inconsistency, which can help identify the specific annotations and taxon constraints that conflict.

When evaluating model organism annotations specifically, we excluded annotations with evidence code ISS. Our analysis revealed that ISS annotations, which are computationally derived (using sequence or structural similarity) rather than experimentally validated or manually reviewed, accounted for the majority of taxonomic consistency violations in curated databases.

### Logical formulation

This subsection consolidates the formal logical foundation of our evaluation framework. It collects the notation used to express protein–function annotations and the precise logical definitions of completeness, coherence, and consistency. Readers who require only the operational use of these measures may proceed to the next section without loss; this subsection is provided for those who wish to inspect the formal grounding of our metrics.

To formalize the framework, we first establish the logical foundations and notation. Our main aim is to extend the evaluation of protein function predictions from single proteins to entire genomes (or proteomes). We first introduce a set of predicates. Basic types (unary predicates) are *genome(x)*, *protein(x)*, and *function(x)*, which allow us to distinguish between the entities we refer to. The distinction between *genome* and *protein* is introduced to reformulate the function prediction task to a task where functions of multiple proteins belonging to the same genome (or proteome) are predicted. We also use every GO class as a basic type (unary predicate), and assume that all GO classes are subclasses of *function* (i.e. for every GO class *F*, we assume $\forall x (F(x) \rightarrow function(x))$). Here, we do not distinguish between the different sub-ontologies of GO.

Throughout, we refer to specific classes of proteins (e.g. the class of proteins BGAL_ECOLI with UniProt identifier P00722) as individuals, not classes; the main reason is that, here, we do not assert further axioms that pertain to proteins, and therefore can use the protein class as a logical individual. This use can also be justified ontologically and integrated with other treatments of proteins in biomedical ontologies [25]. We use the same approach for genomes. Functions in GO are classes, and we instantiate the functions when we assert that a protein has the function; proteins will have (individual) functions that are instances of some GO class. For example, to assert that the *Eschericia coli* lacZ protein is involved in *beta-galactosidase activity* (GO:0004565), we assert $hF(BGAL{\_}ECOLI, c_{tr}) \land \mbox{GO:0004565}(c_{tr})$, where $c_{tr}$ is an individual instance of the GO class Beta-galactosidase activity (GO:0004565). This approach allows us to distinguish between the GO class and specific instances of that function in particular proteins.

As binary predicates, we use *hF(x,y)* for the “has function” relation between a protein *x* and a function *y*, and the newly introduced *in(x,y)* relation that asserts that protein *x* belongs to genome *y*. We can relate arguments of these two relations to the basic types using two axioms:


\begin{align*} & \forall x \forall y (hF(x,y) \rightarrow protein(x) \land function(y)) \end{align*}

and


\begin{align*} & \forall x \forall y (in(x,y) \rightarrow protein(x) \land genome(y)) \end{align*}

The protein function annotation and prediction problem is commonly formulated as the task of assigning a set of functions (from a collection of protein functions $\mathbb{F}$) to a protein *p* from a collection of proteins $\mathbb{P}$, i.e. to find (or learn) a function $f_{hF}$ such that $f_{hF}:\mathbb{P} \mapsto \mathcal{Pow}(\mathbb{F})$, where $\mathcal{Pow}(\mathbb{F})$ indicates the powerset of $\mathbb{F}$, i.e. the set of all subsets of $\mathbb{F}$. The protein $p \in \mathbb{P}$ for which functions are assigned is given by an amino acid sequence, structure, or other feature, and the collection of functions $\mathbb{F}$ is taken from an ontology such as GO, but could also refer to another vocabulary or knowledge base, such as EC numbers (enzyme commission) [26], pathways from KEGG [27], functional domain annotations from InterPro [28] or Pfam [29], or antibiotic resistance annotations [30].

This traditional formulation treats proteins as isolated entities; however, proteins exist within the context of genomes where functional and evolutionary constraints can provide additional context. If whole genomes are available, and the relation between proteins and the genomes that code for them are known, the prediction task can be formulated differently: given the set of genomes $\mathbb{G}$, the set of all proteins $\mathbb{P}$, and a set of protein functions $\mathbb{F}$, find or learn a function $g_{hF}$ such that $g_{hF}: \mathbb{P} \times \mathbb{G} \mapsto \mathcal{Pow}(\mathbb{F})$. In our framework, the tuples $\mathbb{P} \times \mathbb{G}$ are captured by the *in* relation. This genome-centric reformulation of protein function assignment enables us to make use of contextual information about the genomic environment in which proteins operate (the cell or organism) during evaluation.

With this notation in place, the three measures presented in the preceding subsections can be defined as follows.

Definition 1 (Completeness) Let *g* be a genome, let *p* be a protein, and let *F* be a required function from a set of required functions $\mathcal{R}$ that are deemed essential for viability. A genome annotation is complete with respect to $\mathcal{R}$ if and only if for every genome *g* and required function $F \in \mathcal{R}$, there exists at least one protein *p* that is in *g* and that has function *F*. Formally, we assert for every required function $F \in \mathcal{R}$:

\begin{align*} & \forall g ( genome(g) \implies \exists p \exists c_{F} (in(p, g) \land F(c_{F}) \land hF(p, c_{F}))) \end{align*}

Definition 2 (Coherence) Let *g* be a genome, let $P = \{ p | in(p, g)\}$ be the set of proteins in *g*, and let $\mathcal{C}\subseteq \mathbb{F} \times \mathbb{F}$ be a set of pairs of functions (which we call *dependent functions*). We call the set $hF(P) = \{(p, f) | hF(p,f) \land p \in P \}$ of function annotations for proteins in *P*  *genome-level coherent* with respect to $\mathcal{C}$ if and only if the following condition is satisfied for every pair $(F_{1}, F_{2}) \in \mathcal{C}$:

\begin{align*} \forall p_{1} (in(p_{1},g) \land &\exists f_{1} (hF(p_{1},f_{1}) \land F_{1}(f_{1})) \rightarrow \\& \exists p_{2} \exists f_{2}(in(p_{2},g) \land F_{2}(f_{2}) \land hF(p_{2},f_{2}))) \end{align*}

We call the set $hF(P) = \{(p, f) | hF(p,f) \land p \in P \}$ of function annotations for proteins in *P*  *protein-level coherent* with respect to $\mathcal{C}$ if and only if the following condition is satisfied for every pair $(F_{1}, F_{2}) \in \mathcal{C}$:

\begin{align*} \forall p_{1} (in(p_{1},g) \land &\exists f_{1} (hF(p_{1},f_{1}) \land F_{1}(f_{1})) \rightarrow \\& \exists p_{2} \exists f_{2}(in(p_{2},g) \land F_{2}(f_{2}) \land hF(p_{2},f_{2})) \land p_{1} = p_{2}) \end{align*}

From these definitions it is immediate that protein-level coherence implies genome-level coherence but not the other way around. The set $\mathcal{C}$ of dependent functions can be obtained from axioms of GO itself, both for protein-level and genome-level coherence. The empirical evaluations in the preceding subsections focus on the genome-level case; the protein-level form is satisfied by construction when the GO “true path rule” is applied to annotations [14].

Definition 3 (Consistency) Let *g* be a genome, let $P = \{ p | in(p, g)\}$ be the set of proteins in *g*, and let $\mathcal{M}\subseteq \mathbb{F} \times \mathbb{F}$ be a set of pairs of functions (which we call *mutually exclusive functions*). We call the set $hF(P) = \{(p, f) | hF(p,f) \land p \in P \}$ of function annotations for proteins in *P*  *genome-level consistent* with respect to $\mathcal{M}$ if and only if the following condition is satisfied for every pair $(F_{1}, F_{2}) \in \mathcal{M}$:

\begin{align*} \forall p_{1} (in(p_{1},g) \land &\exists c_{F_{1}} (hF(p_{1},c_{F_{1}}) \land F_{1}(c_{F_{1}})) \rightarrow \\& \neg \exists p_{2} \exists c_{F_{2}}(in(p_{2},g) \land F_{2}(c_{F_{2}}) \land hF(p_{2},c_{F_{2}}))) \end{align*}

We call the set $hF(P) = \{(p, f) | hF(p,f) \land p \in P \}$ of function annotations for proteins in *P*  *protein-level consistent* with respect to $\mathcal{M}$ if and only if the following condition is satisfied for every pair $(F_{1}, F_{2}) \in \mathcal{M}$:

\begin{align*} \forall p_{1} (in(p_{1},g) \land &\exists c_{F_{1}} (hF(p_{1},c_{F_{1}}) \land F_{1}(c_{F_{1}})) \rightarrow \\& \neg \exists p_{2} \exists c_{F_{2}}(in(p_{2},g) \land F_{2}(c_{F_{2}}) \land hF(p_{2},c_{F_{2}})) \land p_{1} = p_{2}) \end{align*}

## Datasets and function prediction methods

### Bacterial genome dataset

To evaluate protein function annotations, we selected randomly 1000 complete, annotated genomes (representing *∼*0.5% of available complete bacterial genomes at the time of selection) and their corresponding protein sequences and downloaded them from the NCBI Genomes database [31] on 1 June 2024. We selected genomes from NCBI based on these criteria: assembly level “complete”; taxon: “bacteria (eubacteria)”; annotation: “annotated by NCBI RefSeq.” These genomes represent 1000 distinct species and 556 genera, with an average of 3197 protein-coding genes per genome.

### Gene ontology

Gene functions are described using the GO [1, 14, 32]. GO is a structured, controlled vocabulary that describes the functions of gene products and relationships between functions across all life. It consists of three interconnected sub-ontologies that describe different aspects of biological function: Molecular Function (MF), Biological Process (BP), and Cellular Component (CC). The MF ontology describes specific activities of gene products at the molecular level, such as enzyme catalysis, binding interactions, or transporter functions; functions in MF can be determined based on information about a single proteins. The BP ontology represents functions that require multiple molecular activities working together, like cell division, immune response, or metabolic pathways; they will usually involve more than a single protein. The CC ontology characterizes the location where gene products are active within cells or in the extracellular environment, including organelles, protein complexes, and cellular structures. Here, we use all three branches of GO collectively as “function,” and “function annotation” or “function prediction” to mean the assignment of a GO class to a protein.

There are three main version of GO. GO-basic is a simplified version containing only within-ontology relationships, i.e. no axioms that cross between different sub-ontologies of GO. GO-basic is intended to be sufficient for most biological applications of GO, including gene set enrichment analysis [33]. GO is the core version and includes axioms involving multiple relation types (“part of,” “regulates,” etc.) and allows relationships to cross between different sub-ontologies. GO-Plus is an extended version of GO that contains additional axioms that link to other ontologies [33], such as ChEBI [34] for chemical entities or the Celltype Ontology [35].

For our experiments, we used the GO and GO-Plus (r16-03-2025) in Web Ontology Language (OWL) [36] format. The version of GO we used includes 51 676 classes and 90 088 logical axioms, and GO-Plus includes 84 186 classes and 1004 038 axioms, of which 297 536 are logical axioms. For our evaluation, we relied on GO-Plus axioms involving relations such as “has part” (RO:0000051) and “occurs in” (RO:0000066).

### GO function prediction methods

We annotated protein sequences derived from each assembly using DeepGOMeta (v1) [37], InterProScan (v5.61-93.0) [6], SPROF-GO (r05-12-2022) [38], DeepFRI (v1) [39], TALE (r03-2021) [40], and DeepGraphGO (r07-2021) [41], and the NCBI Prokaryotic Genome Annotation Pipeline (PGAP) (v6.7 and v6.10) [42]. We selected these methods to represent diverse approaches: structural information (DeepFRI), sequence patterns (TALE, DeepGOMeta, SPROF-GO), domain signatures (InterProScan, PGAP), and protein networks (DeepGraphGO).

As DeepFRI can predict functions from Protein Data Bank (PDB) [43] structure files, we mapped RefSeq [31] protein identifiers to UniProt identifiers using the mappings provided by the UniProt Knowledgebase (r02-10-2024) [13]. We then used these UniProt identifiers to download protein structures from the AlphaFold Protein Structure Database (accessed 30 October 2024) [44]. We predicted structures for proteins without available entries in the database or corresponding UniProt identifiers using ESMFold (v1.0.3) [45]. We split proteins exceeding 1024 amino acids into the minimal number of nonoverlapping chunks, and used each chunk separately for structure prediction, according to the recommendations of the ESMFold developers. We then used the resulting structure (PDB format) files for the proteins in an assembly as input for DeepFRI predictions. For proteins containing multiple chunks, we concatenated the resulting annotations from all the chunks belonging to the same protein. For TALE, we split proteins exceeding 1000 amino acids into the minimal nonoverlapping number of chunks, annotated all the chunks, and concatenated the annotations of all the chunks for each protein.

With the exception of InterProScan and PGAP, the methods provide a confidence score for each prediction. For evaluation, we used a prediction score threshold that maximized the protein-centric $F_{\max }$ measure [9] based on a time-based split of the UniProt/SwissProt database (v2023-03, r28-06–2023 to v2023-05 r08-11-2023), following the time-based evaluation procedures established by the CAFA challenge [8]. We obtained a distinct threshold for each GO sub-ontology and for each method, with the exception of InterProScan and PGAP, which do not output a prediction score. For InterProScan and PGAP, we retained all resulting annotations without any filtering. InterProScan maps to GO through curated InterPro-to-GO mappings [46].

We processed annotations resulting from all methods for downstream applications using two distinct strategies. For the first strategy, we only retained the most specific predicted GO classes for each protein by excluding more general ancestor classes (superclasses). In this context, an ancestor encompasses any GO class connected through a “is a,” “part of,” and “occurs in” relationship, as well as any combination thereof. This process involved traversing the GO hierarchy and eliminating any classes that were ancestors of more specific classes already associated with the protein. We followed a second strategy where we expanded the annotations by propagating each protein’s GO classes to include all ancestors in the GO hierarchy.

### Model organism genome annotations

We obtained annotations of six model organisms to test manual function annotations. We obtained annotations of *Mycobacterium tuberculosis* ATCC 25618, *Staphylococcus aureus* NCTC 8325, *Helicobacter pylori* 26695, and *Bacillus subtilis* 168 from the Gene Ontology Annotation (GOA) Database [47]; *E. coli* K-12 from EcoCyc [48]; and *Pseudomonas aeruginosa* PAO1 from the Pseudomonas Genome Database [49] (all databases accessed 12 March 2025). We selected these six organisms as they represent well-studied bacterial models with extensive manual curation across diverse lifestyles: free-living (*E. coli, B. subtilis*), pathogenic (*S. aureus, H. pylori, M. tuberculosis*), and opportunistic (*P. aeruginosa*). We retained annotations with the following evidence codes: experimental: EXP, IDA, IPI, IMP, IGI, IEP; high-throughput experimental: HTP, HDA, HMP, HGI, HEP; computationally derived: ISS, ISO, ISA, IGC; curator or author statement: TAS, NAS, IC; and electronic: IEA.

#### Information content

We calculated the information content (IC) of GO classes to measure the specificity of functional annotations based on Resnik’s formulation [50]. We computed the IC of a GO class *c* for a given genome *g* as


\begin{align*}& \mathrm{IC}(c,g) = -\log(p(c,g)), \end{align*}

where *p(c,g)* is the relative frequency of class *c* in the set of all GO classes annotated to proteins in genome *g*. This formulation ensures that IC reflects the specificity of GO classes within the context of each genome, where frequently occurring (general) classes receive lower IC values, while less frequent (specific) terms receive higher IC values.

To summarize annotation specificity, we computed two metrics per genome: IC depth, defined as the mean IC across all annotations in a genome, representing the average specificity of the predicted functions; and IC breadth, defined as $\sum _{c \in C_{g}} IC(c,g)$, where $C_{g}$ is the set of all GO classes annotated in *g*. To account for differences in genome size, we calculated a normalized IC breadth value by dividing the total IC by the number of proteins in the genome to capture the total functional specificity assigned per protein.

## Genome-scale evaluation of function annotations

### Manually curated function annotations

**Figure 1:**
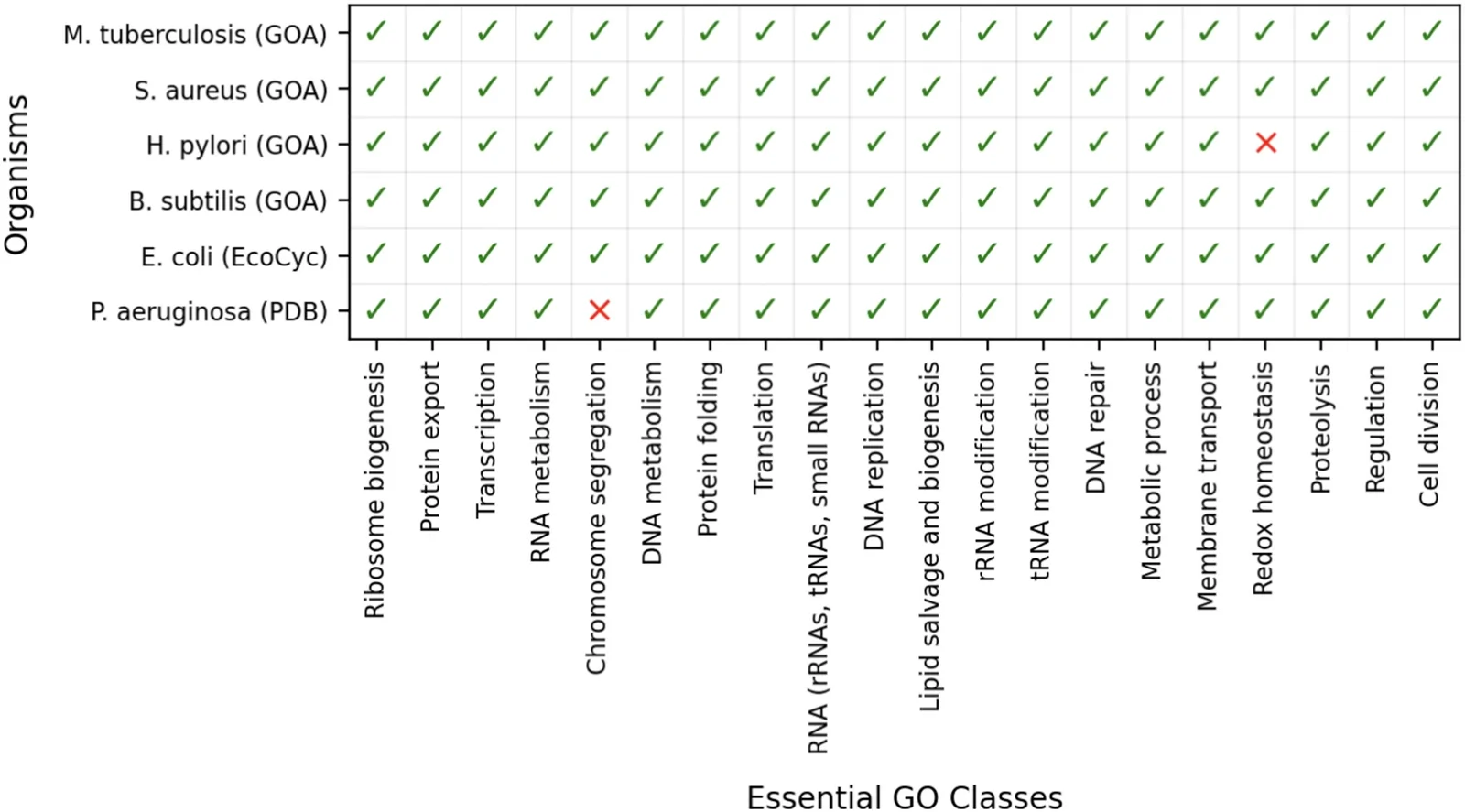
Presence or absence of “Core” GO classes in genome annotations from model organisms, with checked cells indicating presence of at least one annotation to the corresponding class.

We first established baseline expectations by evaluating annotations from well-characterized model organisms. We evaluated the completeness of essential functional categories in model organism annotations (Fig. 1). All essential function classes were present in all model organism annotations, with the exception of *Chromosome segregation* (GO:0007059) in *P. aeruginosa* and *Redox homeostasis* (GO:0045454) in *H. pylori*. This high coverage of essential functions validates our choice of well-studied organisms and our selection of essential functions.

Next, we evaluated the coherence of model organism annotations using process, pathway, and complex-based dependencies (Table 2). *Eschericia coli* had the highest coherence across all measures, reflecting that it is one of the most extensively studied bacterial model organisms [51], and indicating that coherence measurements effectively capture the completeness of our functional knowledge rather than annotation quality.

**Table 2:** Percentage of coherent processes, MetaCyc pathways, and protein complexes across selected model organisms.

Model organism	Processes	Pathways	Complexes
*B. subtilis*	94.00%	61.84%	63.51%
*E. coli*	96.25%	82.84%	84.23%
*H. pylori*	92.77%	45.10%	73.21%
*M. tuberculosis*	95.98%	61.45%	72.97%
*P. aeruginosa*	83.90%	20.83%	72.73%
*S. aureus*	93.29%	48.39%	65.67%

Among different coherence measures, process coherence was the highest value across all organisms. One possible explanation is that “has part” relationships are explicitly encoded in GO’s ontological structure, whereas pathway and complex coherence information is not; therefore, curators are aware of “has part” dependencies when making annotations but not of other dependencies. Consequently, coherence metrics capture annotation completeness and implicit biological knowledge across curation approaches. *Eschericia coli*’s higher coherence score reflects intensive research, and high process coherence across organisms demonstrates the effectiveness of GO’s axiomatic structure in maintaining functional dependencies.

Next, we evaluated taxonomic consistency based on *in taxon* constraints expressed in GO [23]. *Bacillus subtilis* and *S. aureus* fully satisfied taxonomic consistency constraints, while the other four genomes contained functions with mutually exclusive taxonomic requirements. We identified three causes of violations.

First, violations of taxon constraints may indicate annotation errors, e.g. in *P. aeruginosa*, the protein UniProt:PA5451 is annotated with *Pellicle* (GO:0020039), which has a “never in taxon” *Bacteria* constraint. Although the annotation is manually curated, the referenced publication does not mention the pellicle, suggesting a potential problem in the annotation process and motivating automatic semantic checks (as implemented in our evaluation framework). Second, an annotation’s taxonomic constraint in GO may be overly restrictive. In *E. coli*, the protein UniProt:P60723 is annotated with *RNA-binding Transcription Regulator Activity* (GO:0001070), which has the “only in taxon” *Viruses* constraint. However, the definition of this class describes a regulatory mechanism that is also known to occur in *E. coli* [52]. Third, some annotations describe functions of pathogens within a host. For example, in *H. pylori* and *M. tuberculosis*, proteins UniProt:O25743 and UniProt:P9WQP1 are annotated with *Negative regulation of T cell proliferation* (GO:0042130) [53] and *Positive regulation of plasminogen activation* (GO:0010756) [54], respectively. Both proteins are correctly annotated with these functions yet perform them only during infection, and the functions (biological processes) occur in the host, not in the organisms themselves.

**Figure 2:**
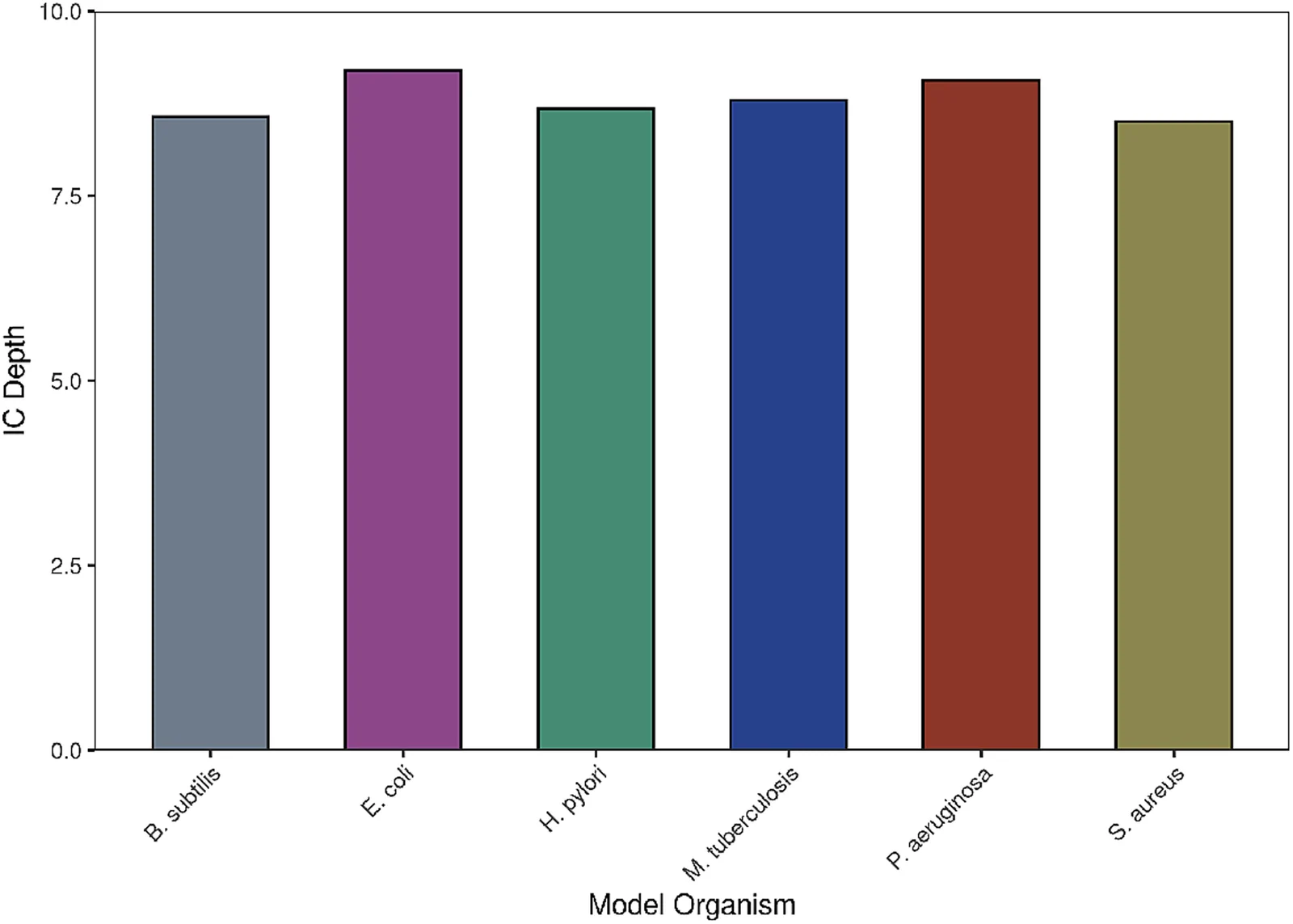
IC depth for specific GO class annotations across six bacterial model organisms: *E. coli, B. subtilis, P. aeruginosa, H. pylori, S. aureus*, and *M. tuberculosis.*

**Figure 3:**
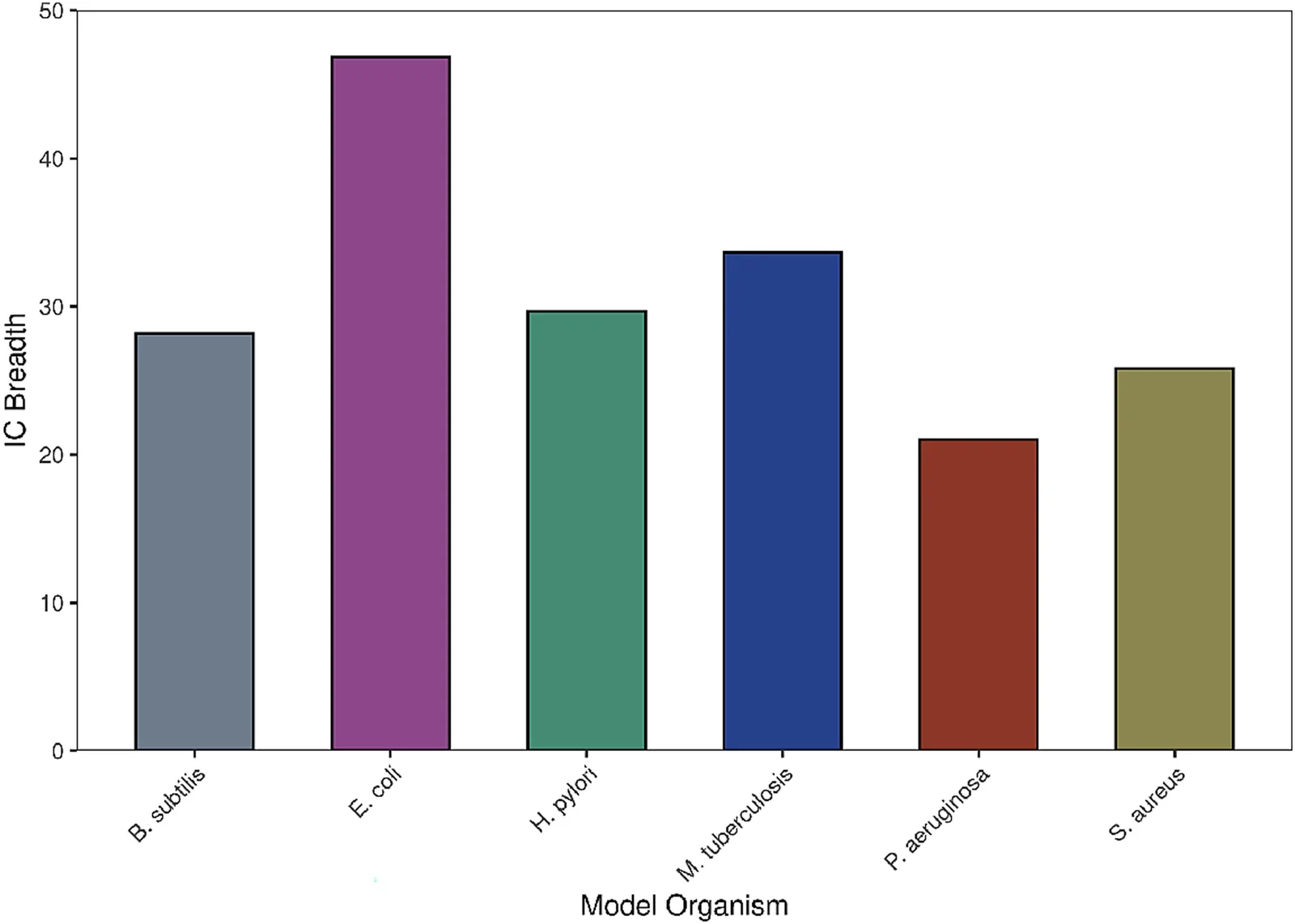
IC breadth normalized by the number of proteins for specific GO class annotations across six bacterial model organisms: *E. coli, B. subtilis, P. aeruginosa, H. pylori, S. aureus*, and *M. tuberculosis.*

Beyond structural constraints, we assessed the specificity of GO annotations using the average IC of asserted annotations (see Fig. 2). Model organism annotations demonstrated high average IC, with *P. aeruginosa* and *E. coli* the highest (i.e. the annotations have the highest average IC). We also computed IC breadth to quantify the sum of all the functional information per protein (Fig. 3). *Eschericia coli* was the highest, and *P. aeruginosa* the lowest, indicating a depth–breadth tradeoff (deep–narrow versus broad–shallow). These findings suggest that annotation specificity and systematic coherence represent complementary rather than competing aspects of annotation quality.

### Automated function annotations

Having established baseline performance from model organism annotations, we evaluated seven computational methods representing distinct function prediction approaches. These methods leverage different biological information sources: structural data (DeepFRI [39]), primary sequence patterns (TALE [40], DeepGOMeta [37], SPROF-GO [38]), protein domain signatures (InterProScan [6], PGAP [42]), and protein interaction networks (DeepGraphGO [41]). This diversity allows us to assess how different types of biological evidence contribute to genome-scale annotation.

**Figure 4:**
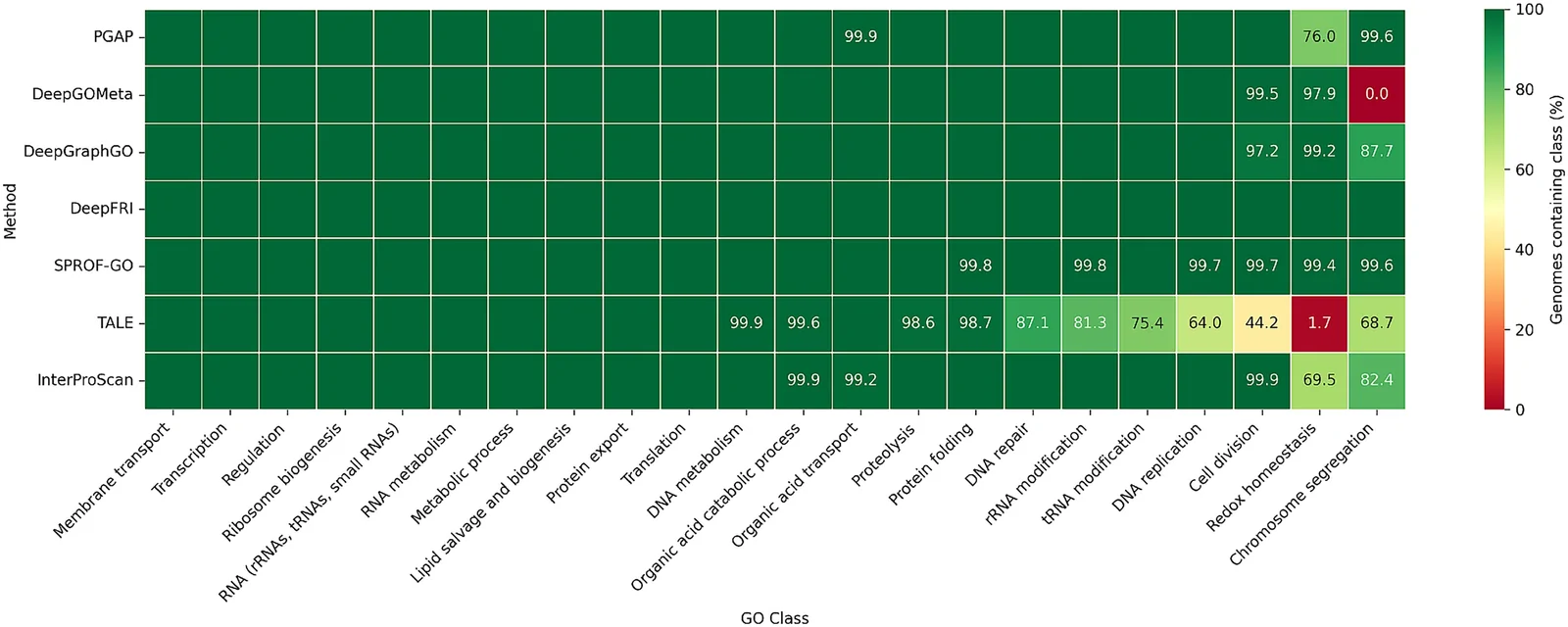
Evaluation of essential GO class presence in bacterial genomes annotated using several methods. Numeric labels highlight <100% presence.

We applied each method to 1000 bacterial genomes, and evaluated the genome-scale results. Our analysis reveals systematic differences in how methodological approaches perform across the evaluation criteria. When evaluating essential function completeness (Fig. 4), using structural information was most effective: DeepFRI detected all 22 “Core” GO classes in 100% of genomes. This may reflect the direct relationship between protein structure and essential functions. In contrast, sequence-based methods had variable performance, as TALE had the lowest coverage, particularly for *Redox Homeostasis* (GO:0045454) at 1.7% and *Cell Division* (GO:0051301) at 44.2%. *Chromosome Segregation* (GO:0007059) was systematically underpredicted across methods, with DeepGOMeta failing to annotate it entirely. This pattern mirrors gaps observed in model organism annotations, suggesting challenges in recognizing these functions across both computational and manual approaches.

**Figure 5:**
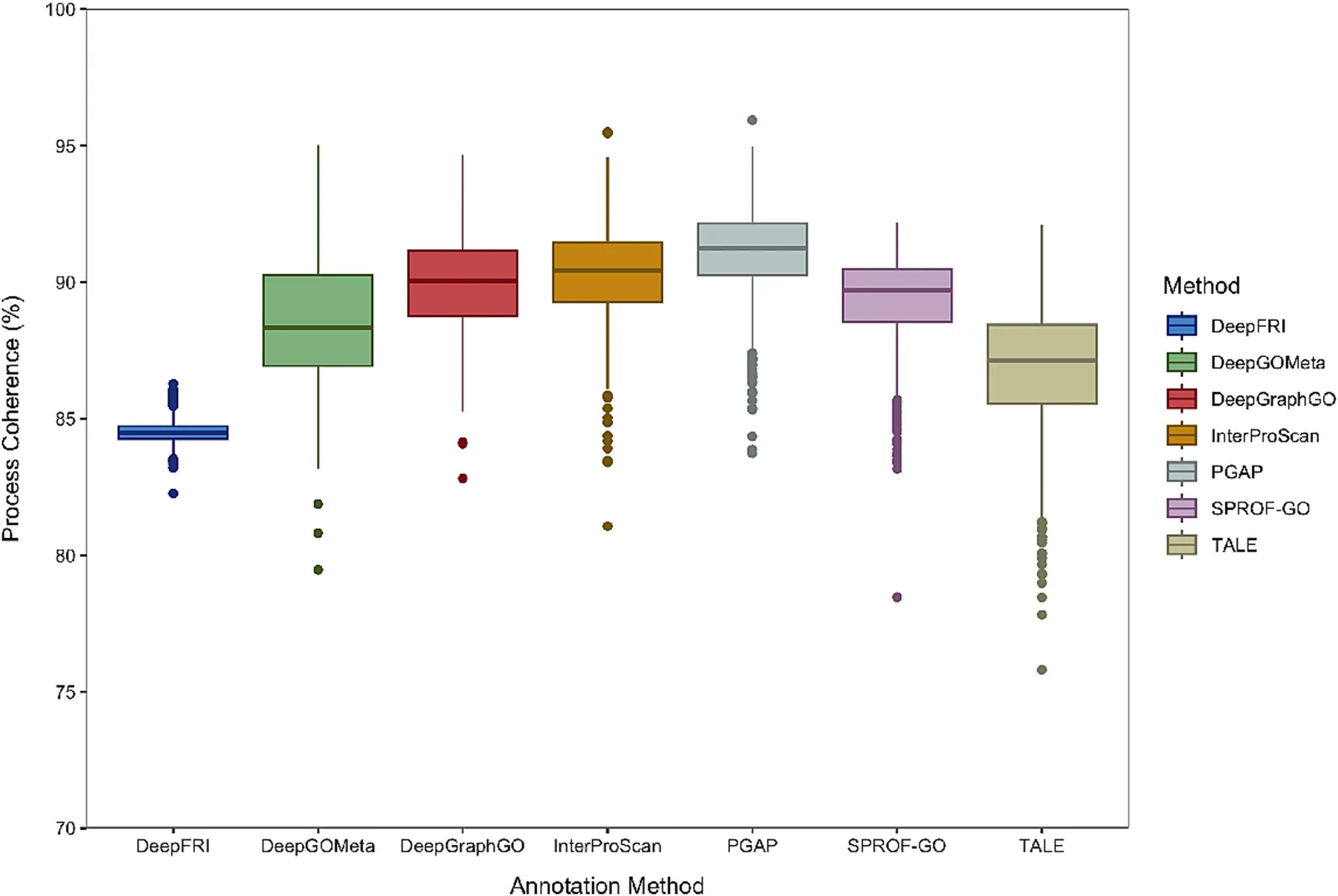
Distribution of process coherence for each method based on the percentage of missing “has part” (RO:0000051) relations in the annotations.

Coherence measurements reveal how different methodological approaches capture functional dependencies. We measured process coherence by finding “has part” relation in GO, and, if process *P* has *Q* as part and some protein *p* is annotated with *P*, determine whether there is a protein $p^{\prime}$ in the same genome that is annotated with *Q* (Fig. 5). Methods incorporating ontological structure, protein–protein interactions, or domain knowledge (DeepGraphGO, InterProScan, PGAP) achieved performance comparable with model organism annotations. DeepFRI had the lowest process coherence. Our results show that using ontological structure is important in function prediction, independent of the features used.

**Figure 6:**
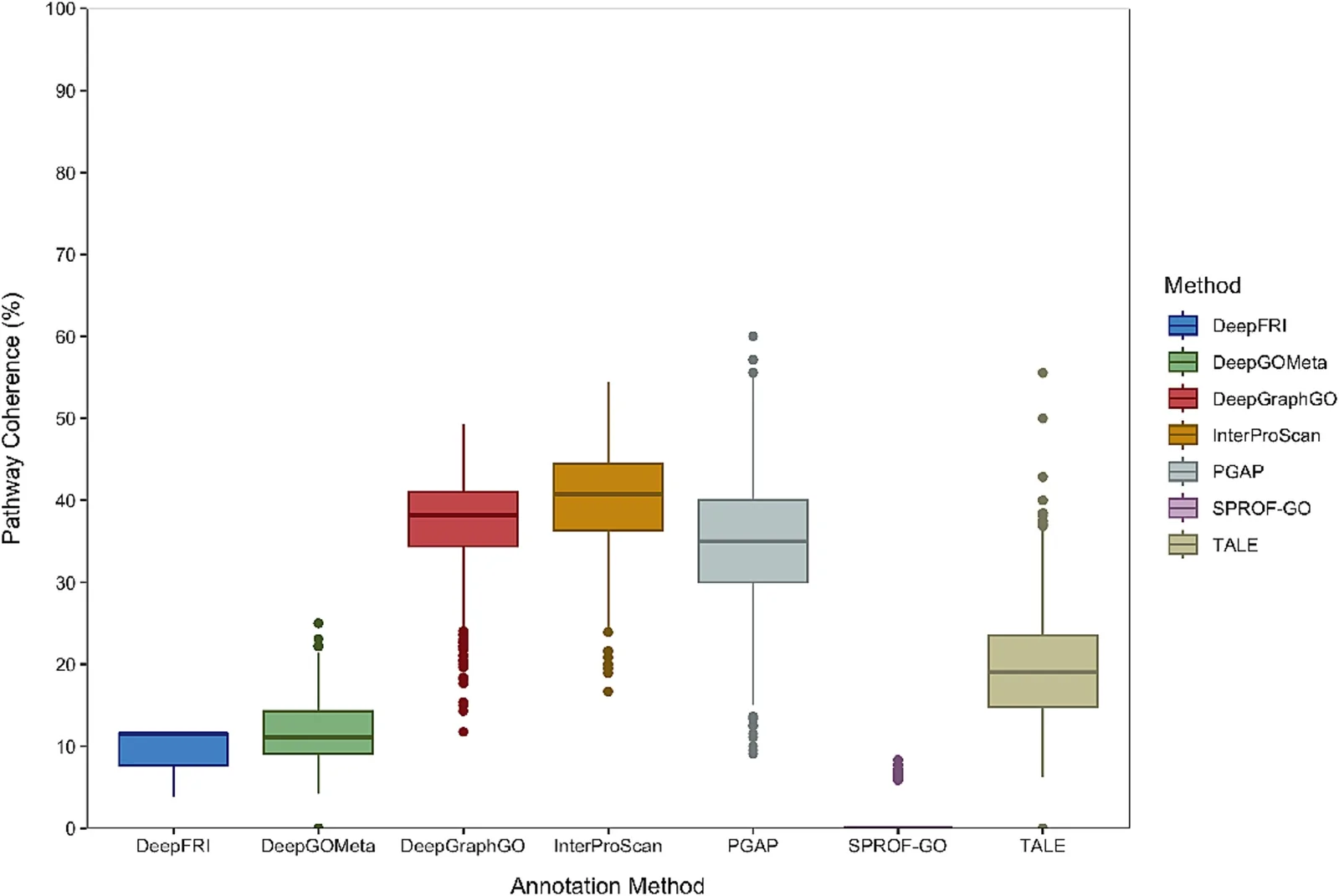
Distribution of pathway coherence for each method based on the percentage of complete MetaCyc pathways in the resulting annotations.

Pathway coherence measures whether annotated pathways are complete within a genome (Fig. 6). Methods based on interaction networks (DeepGraphGO) and domains (InterProScan, PGAP) are better at recovering complete metabolic pathways, similarly to curated function annotations such as *H. pylori* and *S. aureus*. For sequence-based methods, SPROF-GO failed to complete any pathways, and TALE performed better than other sequence-only approaches, suggesting that it is not only the used features but also model training that affects performance.

**Figure 7:**
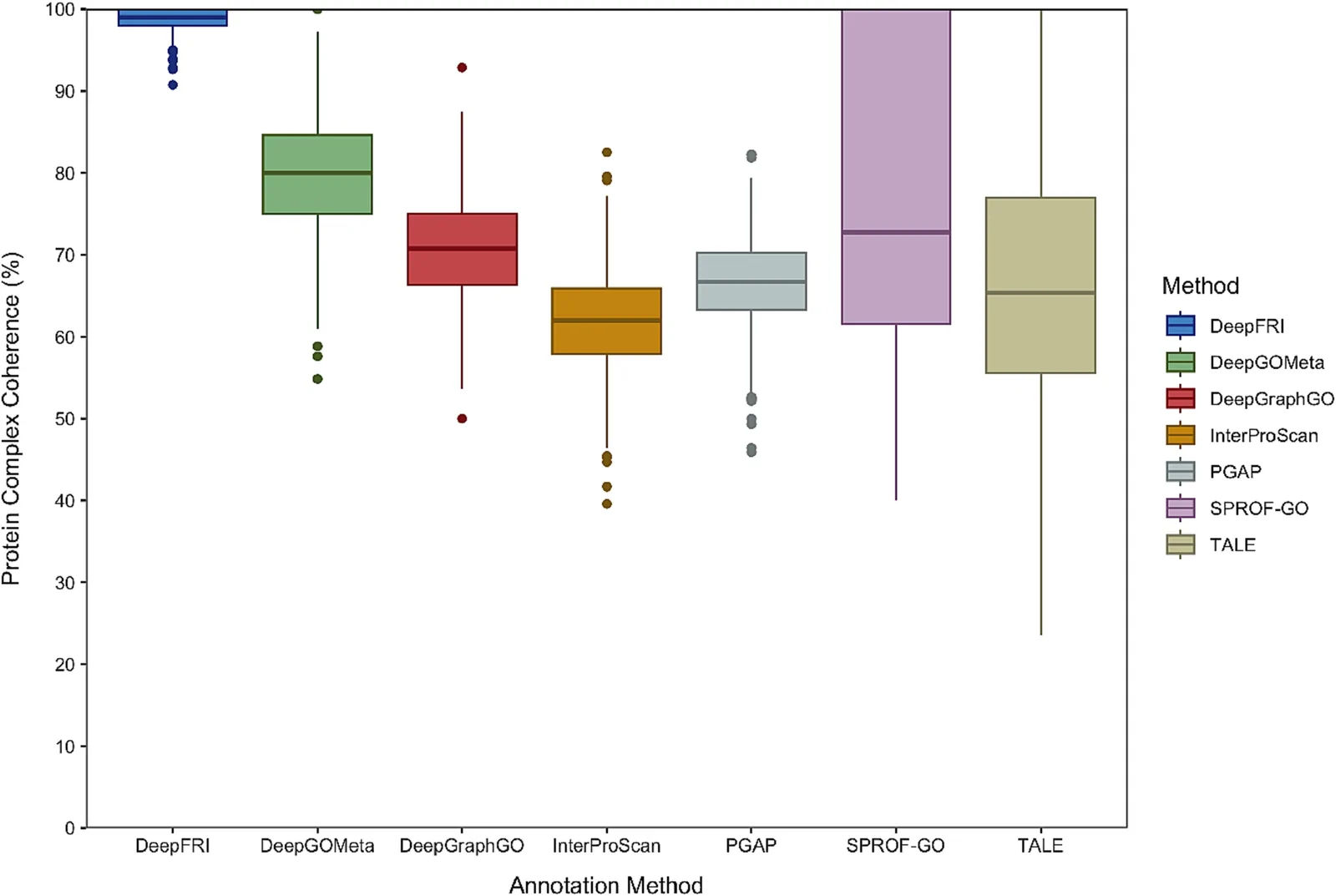
Distribution of protein complex coherence based on GO protein-protein complex annotations for each method.

Complex coherence is determined based on whether there is more than one protein in a genome that is annotated with the same heteromeric complex, i.e. whether the parts of the protein complex are known (Fig. 7). DeepFRI achieved the highest coherence with minimal variation, even exceeding *E. coli* performance. This reflects the direct structural basis of protein–protein interactions in complex assembly. Surprisingly, InterProScan and PGAP showed some of the lowest average complex coherence, suggesting that domain signatures alone inadequately capture the interactions required for complex formation.

We evaluated taxonomic consistency by testing whether predicted GO annotations violated curated “only in taxon” (RO:0002160) or “never in taxon” (RO:0002161) constraints in the GO. InterProScan annotated 361 of the 1000 genomes in a taxonomically consistent manner, whereas all other methods did not provide any taxonomically consistent sets of annotations. We identified multiple patterns of constraint violations. Most methods frequently violated constraints involving virus-associated GO classes such as *Virion Component* (GO:0044423), *Virus Tail* (GO:0098015), and *Viral Capsid Assembly* (GO:0019069), which carry “never in taxon” constraints for cellular organisms or bacteria.

Additionally, many violations involved organism-specific constraints. TALE annotated archaeal enzymatic processes, and DeepGraphGO and SPROF-GO annotated with classes restricted to Viridiplantae, involving plant-specific cellular components and processes such as *Apoplast* (GO:0048046) and *Plant Organ Development*.

Other frequent taxonomic consistency violations involved multicellular organismal processes incompatible with bacterial genomes. DeepGOMeta primarily annotated terms related to immune processes, such as *Cytokine Production* (GO:0001816), while DeepGraphGO often annotated animal-specific processes like *Apoptotic process* (GO:0006915), which is restricted to Opisthokonta.

### Annotation specificity

**Figure 8:**
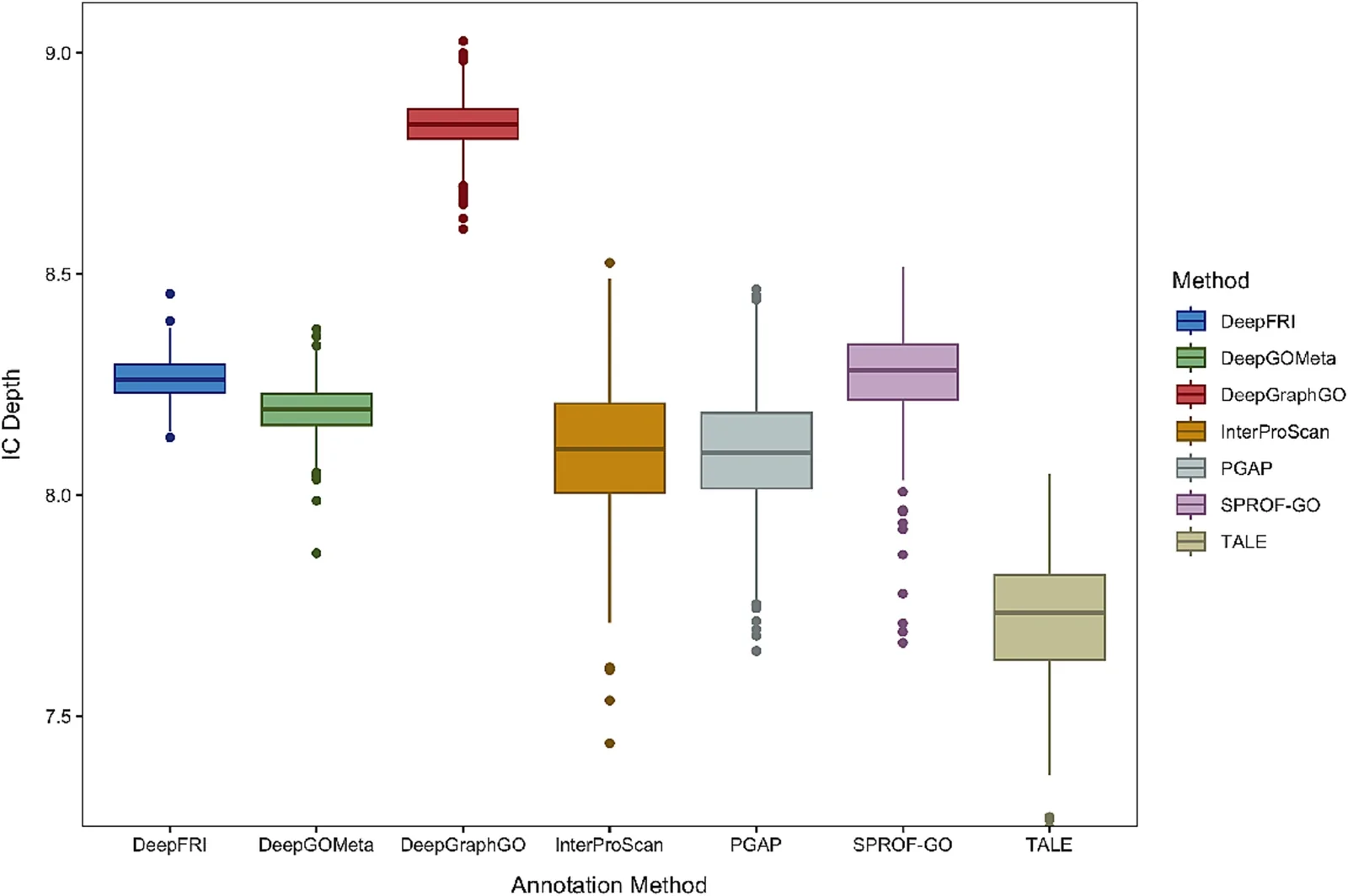
Distribution of IC depth for specific GO classes across the seven methods.

The evaluated methods varied in annotation specificity measured by average IC (Fig. 8). The network-based method DeepGraphGO produced the most specific annotations, similarly to *E. coli* and *P. aeruginosa*, the model organisms with the highest average IC. Other methods, except TALE, achieved functional specificity comparable with other model organisms. This indicates that while these methods may lack coherence (i.e. they do not completely satisfy functional dependencies), they nevertheless identify very specific functional assignments.

**Figure 9:**
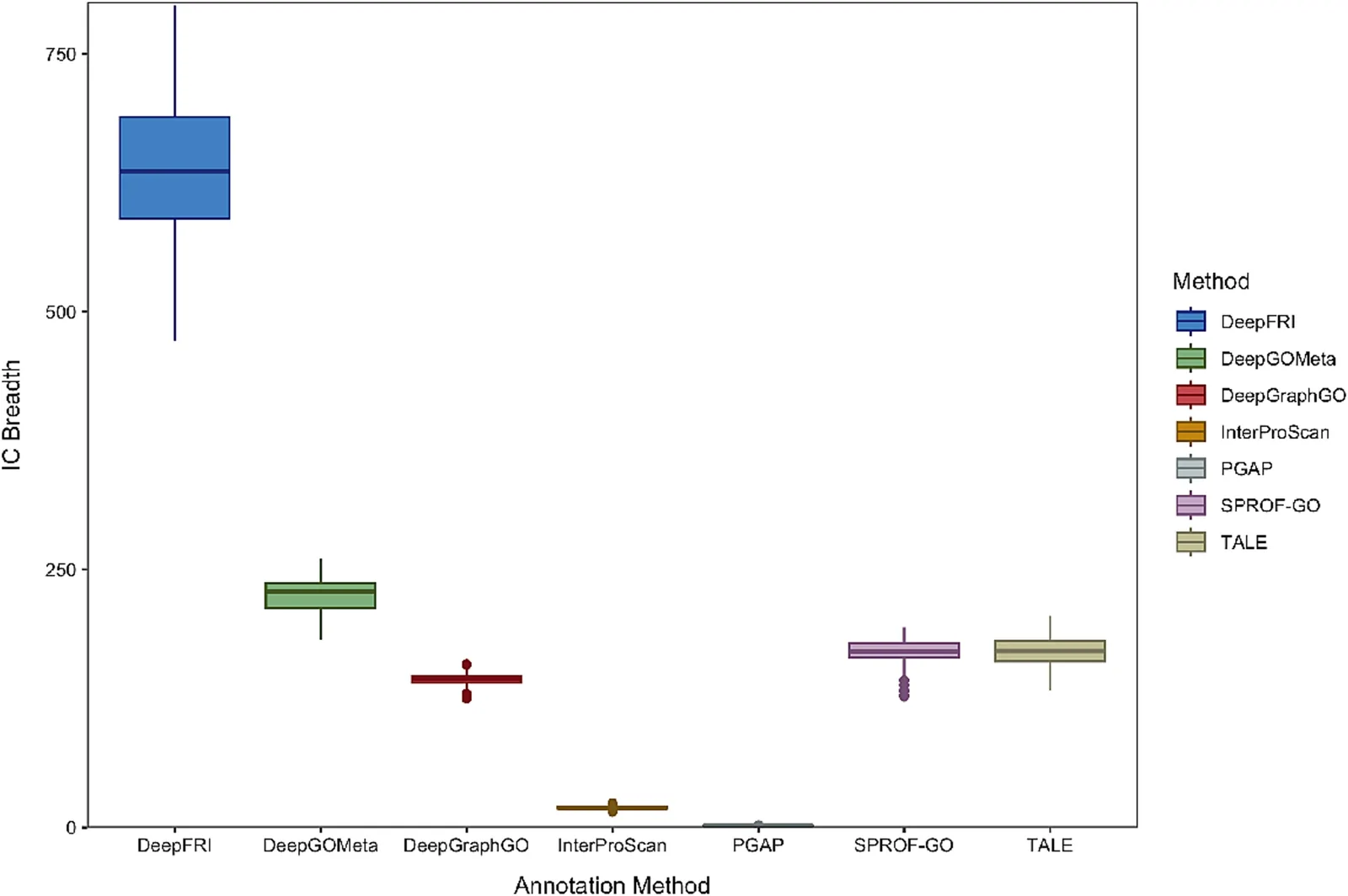
Distribution of IC breadth for specific GO classes across the seven methods.

For total IC per protein, measured by IC breadth (Fig. 9), DeepFRI had higher IC breadth in comparison with all methods and model organisms. The sequence-based methods DeepGOMeta and SPROF-GO also showed high breadth, while InterProScan sat at the low end of the model-organism range and PGAP produced the most conservative annotations of any method evaluated. This indicates a fundamental difference between manual curation (conservative, high-confidence) and automated prediction (broad, lower-confidence) strategies, with domain- and curation-based methods at the conservative extreme.

### Comparison with the CAFA evaluation

While our three evaluation criteria assess the biological plausibility of genome-scale annotations, it remains an open question how these system-level metrics relate to existing benchmarks that focus on individual protein accuracy. To answer this question, we examined the relationship between our framework and other benchmarking approaches. The CAFA [8] currently represents the gold standard for evaluating protein function prediction methods. To understand how our genome-scale evaluation framework relates to this established benchmark, we performed a systematic comparison between our three metrics (completeness, coherence, consistency) and CAFA’s performance measures.

CAFA evaluates methods using $F_{max}$ (maximum F-score across all prediction thresholds) and $S_{min}$ (minimum semantic distance) [9], both computed on held-out test proteins. These metrics capture prediction accuracy at the individual protein level. We tested whether methods optimized for CAFA performance also produce complete, coherent, and consistent genome-scale annotations.

To test this hypothesis, we computed CAFA metrics for five prediction methods (DeepFRI, DeepGOMeta, DeepGraphGO, TALE, and SPROF-GO) using a time-based split of UniProt/SwissProt (training: v2023-03, r28-06-2023; test: v2023-05, r08-11-2023); see Table 3. This temporal split mimics CAFA’s evaluation strategy where methods are tested on proteins annotated after training. We did not include InterProScan or PGAP in the evaluation as they do not provide confidence scores to compute threshold-dependent metrics.

**Table 3:** Threshold that maximized $F_{\max }$ for each GO sub-ontology for each method.

Method	MFO	CCO	BPO
SPROF-GO	0.13	0.54	0.13
DeepGOMeta	0.27	0.27	0.11
TALE	0.28	0.56	0.15
DeepFRI	0.28	0.01	0.01
DeepGraphGO	0.33	0.21	0.30

For correlation analysis, we matched evaluation contexts: when comparing completeness (which uses BPO terms), we correlated against $F_{max}$ computed specifically for BPO. Similarly, we compared protein complex coherence against CCO-specific metrics, and pathway coherence against BPO metrics (as metabolic pathways are classified under biological processes). We computed Spearman’s rank correlation coefficient [55] to assess whether methods that perform well on CAFA benchmarks also produce annotations that satisfy our genome-scale constraints. This analysis reveals whether the CAFA evaluation paradigm adequately captures the requirements for biologically plausible proteome annotation.

**Figure 10:**
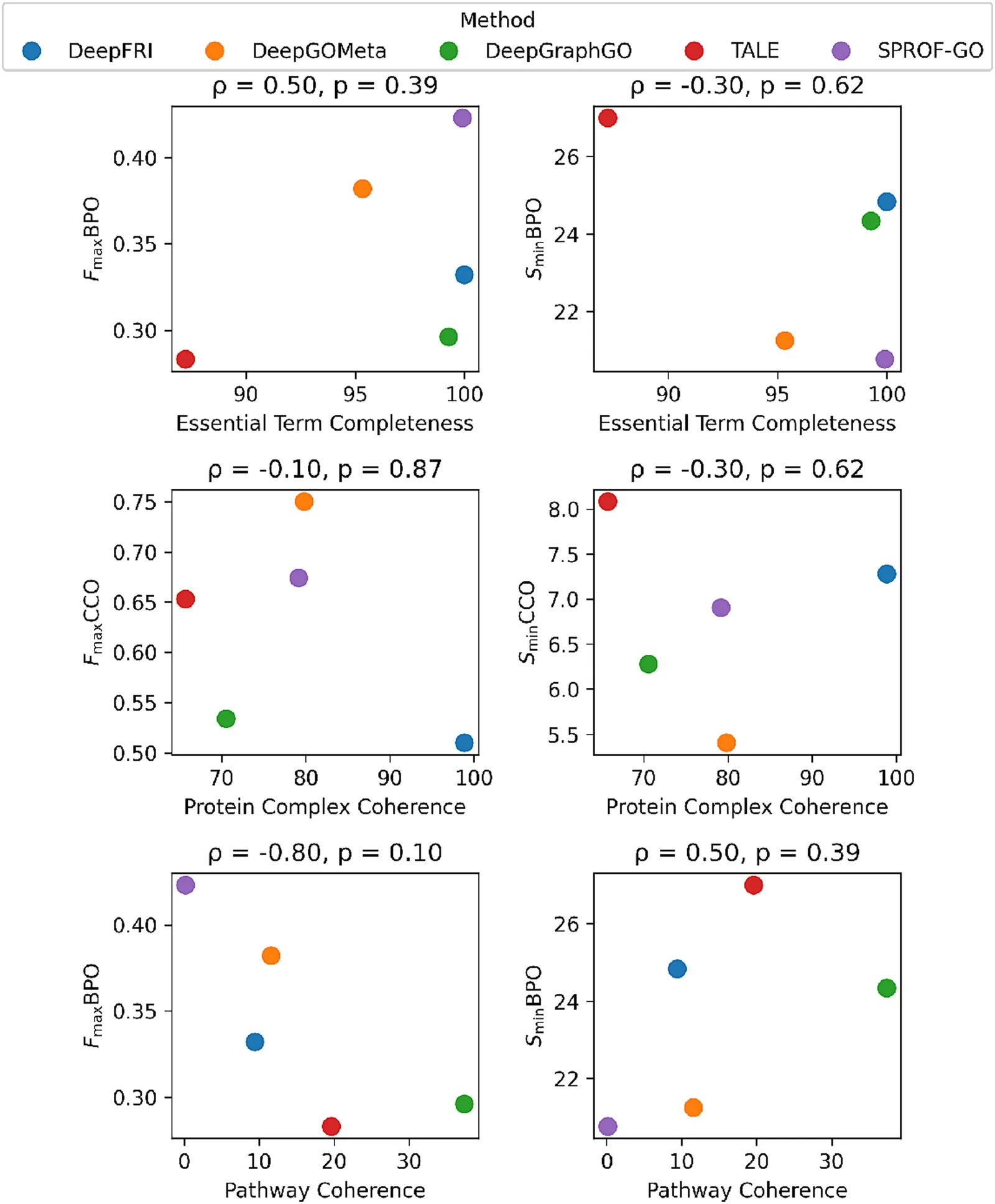
Spearman correlations between evaluation metrics ($F_{\max }$, $S_{\min }$) and framework evaluation metrics (Essential term completeness, protein complex coherence, and pathway coherence).

Figure 10 shows the result, and we find no significant correlation between the CAFA performance and our system-level evaluation. This indicates that our evaluation framework is complementary to the CAFA metrics. $F_{max}$ optimization results in predictions that are specific enough to carry information but general enough to avoid being penalized in a precision-recall trade-off. In contrast, our completeness and coherence metrics require specific terms to fulfill requirements.

## Discussion

Our results show that the protein-level prediction objectives used by current annotation methods do not, on their own, enforce genome-scale completeness, coherence, or consistency. While these methods effectively characterize (either through manual curation or automatic prediction) individual protein functions, the resulting genome-scale annotations are often not biologically coherent. The rationality of our evaluation framework is supported by the performance difference between manually curated model organism annotations and automated prediction methods, demonstrating that the system-level constraints are largely representative of the biological ideals already present in high-quality annotations. Manually curated model organisms mainly showed taxonomic inconsistencies and incomplete pathway annotations, and overall had better system-level metrics compared with automated methods across all three evaluation criteria. Even so, the gaps that remain in manually curated annotations point not to curation errors alone but to limitations of an annotation model designed around individual proteins rather than around the dependencies between their functions.

### Limitations of the reductionist paradigm

Even gold-standard, manually curated databases show systematic gaps in capturing system-level constraints—pathway coherence falls well below process coherence in every model organism we examined, and four of the six show taxonomic inconsistencies. These gaps reflect a structural property of the underlying annotation model: the GO was designed to decompose biological complexity into discrete, manageable units. Developed in the late 1990s when the first eukaryotic genomes became available [2, 14], each gene product receives independent functional labels, as if biology operated through isolated activities rather than integrated systems.

The GO was designed to capture our current biological knowledge as it evolves, not to enforce system-level completeness. For example, annotating a protein with “glycolytic process” when only some pathway enzymes are characterized is appropriate as it accurately reflects our current understanding. However, such annotations carry implicit logical and ontological commitments: they assert that other proteins with complementary functions must exist for the annotated process to occur (even if these proteins are not known). The evaluation framework we establish here may help to make these implicit logical and ontological implications explicit.

The notion of coherence measures the completeness of our current knowledge: when we observe incomplete metabolic pathways in well-studied organisms, it indicates gaps in our understanding rather than errors. Similarly, when computational methods violate system-level constraints, this reveals their failure to capture the ontological dependencies inherent in biological systems. Pathway “hole filling” methods utilize homology and known metabolic pathways to identify missing enzymes [56], providing a key precedent for using systems-level constraints to improve annotations. GO-CAMs [10] explicitly model such multi-protein processes but they are domain-specific and manually curated, and do not express general genome-wide constraints. Similarly, while the SBML Systems Biology Markup Language (SBML) [57] can model biochemical networks, it is not directly connected to GO—reactions can be mapped to GO terms without expressing dependencies [58]. *Post hoc* tools such as SBML Harvester [59] can flag inconsistencies between GO annotations and model structure, but they validate rather than integrate constraints. On the genome quality-control side, tools such as BUSCO [60] and CheckM [61] estimate assembly completeness through the recovery of expected single-copy orthologs, but do not assess whether the functions assigned to encoded proteins describe a viable biological system. The contribution of our evaluation framework is moving toward a logical and ontological language integrated in the GO where conditional dependencies between functions of multiple proteins can be expressed, providing a direct evaluation of an annotation set’s biological plausibility that, to our knowledge, no existing tool delivers.

### Technical limitations and their effects

Technical factors may explain why current methods fail the evaluation criteria we establish. First, the hierarchical structure of GO combined with CAFA’s $F_{max}$ optimization may create a bias toward predicting general, high-confidence terms rather than specific functions. Methods maximize $F_{max}$ by making safe predictions at higher ontology levels, avoiding the risk of incorrect specific annotations. This explains the paradox of high IC breadth but poor pathway completion: methods predict broadly applicable functions while missing the specific enzymes required for complete pathways. This also explains the lack of correlation between CAFA metrics and our system-level metrics; $F_{max}$ is an additive metric, whereas our framework requires specific terms to satisfy constraints. An IC-weighted $F_{max}$ metric [9] could address this by rewarding specific predictions.

Second, the sequence-based data splits used in training function prediction models can create systematic blind spots. Methods trained on sequence similarity-based splits may miss protein families and their functions due to absence from training data. This violates the assumption that training and test distributions share the same label space, leading to functions that can never be predicted regardless of the model. This explains why certain functions show 0% prediction rates across methods despite being essential for life.

### Limitations

We evaluated exclusively on bacterial genomes, while archaea and eukaryotes present distinct challenges through cellular compartmentalization. Furthermore, our genome-centric approach cannot evaluate distributed metabolic systems where essential functions span multiple organisms, such as in syntrophic communities. Future versions of our framework could move from binary logical constraints to probabilistic ones to better accommodate rare biological exceptions or lateral gene transfer events. For example, by assigning a confidence score to taxonomic constraints based on the level of evidence in the GO, the framework could distinguish between “hard” constraints (e.g. eukaryotic-specific organelles) and “soft” constraints that may be subject to revision as our understanding of non-model organisms evolves.

Our essential function set derives from a single minimal genome (Syn1.0), which may not capture alternative life strategies or community-dependent organisms that rely on metabolic complementarity. The pathway analysis depends on MetaCyc coverage, missing organism-specific pathways, and assumes complete pathways exist within single genomes.

Finally, we evaluated methods that are not designed for genome-scale coherence—their low performance reflects optimization for different objectives rather than fundamental algorithmic limitations. Future work should expand to diverse taxonomic groups, develop meta-organism evaluation frameworks, and integrate additional pathway databases, which would require formalizing functional coherence across multiple cooperating genomes rather than single organisms.

### Proposed strategies for improvement

To bridge the gap between protein-level accuracy and system-level coherence, we propose three directions for next-generation prediction models. First, models could move beyond standard cross-entropy or $F_{max}$ optimization and incorporate system-level loss terms that penalize taxonomic inconsistencies or metabolic “holes” during training; this extends a line of work that already uses GO structure to constrain predictions at the protein level (e.g. DeepGOPlus [62]) by pushing the constraints to the genome scale. Second, predictions could be refined *post hoc* by adjusting an initial annotation set so that completeness, coherence, and consistency are jointly satisfied; this generalizes the principle of pathway “hole filling” [56] from missing enzymes in single metabolic pathways to a broader class of GO-expressible constraints. Finally, current single-sequence architectures could be extended to proteome-scale models that operate over a whole organism. Existing graph-based predictors such as DeepGraphGO [41] already incorporate protein–protein interaction networks but still produce per-protein outputs; learning over the entire proteome graph would let models exploit functional dependencies directly during prediction.

## Conclusions

We developed a genome-scale evaluation framework to assess the biological plausibility of protein function annotations based on the principles of completeness, coherence, and consistency, which we introduced and defined. We find that manually curated annotations for model organisms largely satisfy these system-level constraints, suggesting that our criteria are representative of the requirements of a viable biological system. In contrast, we find that current computational prediction methods systematically fail to produce biologically viable proteome-scale annotations. Our results show no significant correlation between individual protein prediction accuracy, as measured by CAFA metrics, and the satisfaction of genome-scale requirements, indicating that our evaluation criteria are complementary to established methods.

The gap we observe is consistent with the design of current annotation pipelines, which optimize accuracy at the level of individual proteins, while completeness, coherence, and consistency are properties of the annotation set as a whole. Our framework operationalizes these three properties as logical constraints over the GO, providing measurable criteria that augment, rather than replace, existing protein-level methods. The three strategies we outline—system-level loss functions, *post hoc* constraint satisfaction layers, and proteome-scale architectures—offer concrete directions for incorporating these criteria into next-generation annotation methods, particularly as the volume of sequenced genomes and metagenomes continues to outpace manual curation.

**Key Points**

- Current protein function annotation methods follow a reductionist paradigm that treats proteins as isolated entities rather than parts of integrated biological systems.
- Three properties measure genome-scale biological plausibility: completeness ensures the presence of essential functions; coherence guarantees that functional dependencies, such as pathway steps and protein complexes, are satisfied; and consistency prevents the co-occurrence of mutually exclusive or taxonomically contradictory functions.
- Manually curated model organism annotations generally satisfy these system-level constraints, but automated prediction methods systematically fail to produce biologically viable genome-scale results.
- There is no significant correlation between individual protein prediction accuracy (CAFA metrics) and the satisfaction of genome-scale requirements, indicating these metrics are complementary.

## Supplementary Material

supp_bbag336

## Data Availability

Data and software generated for this work is available at https://github.com/bio-ontology-research-group/GAEF.
