# Supplementary material for: Evaluating completeness, coherence, and consistency of genome-scale function annotations
Source: Brief Bioinform. 2026 Jun 29;27(3):bbag336. doi: 10.1093/bib/bbag336 (PMC13310775; doi:10.1093/bib/bbag336)
Supplement: supp_bbag336 [file supp_bbag336.pdf]

1. Supplementary Materials

1.1. Annotation Retention Threshold

We used the confidence scores produced by each method to filter the resulting annotations for all methods, with the exception of InterProScan and PGAP, which do not provide confidence scores for the resulting predictions. The threshold at which we retained the annotations is determined as the threshold that maximized $F_{\max}$ [1] in the time-based split evaluation from our previous work on DeepGOMeta [2] (Table S1).

1.2. Essential Function Mappings

We manually mapped functional categories determined to be essential for the survival of *Mycoplasma mycoides* from the Syn1.0 genome [3] to GO classes (Table S2). We were able to map most functional categories directly using exact string matches to the GO class name, definition, or description. We manually reviewed every mapping to ensure accuracy. We could not find a relevant term that directly maps to the functional category 'Transport and catabolism of nonglucose carbon sources' from Syn1.0, so we split it into 'Transport of nonglucose carbon sources' and 'Catabolism of nonglucose carbon sources', and mapped the split categories to the relevant GO classes.

2. Figures

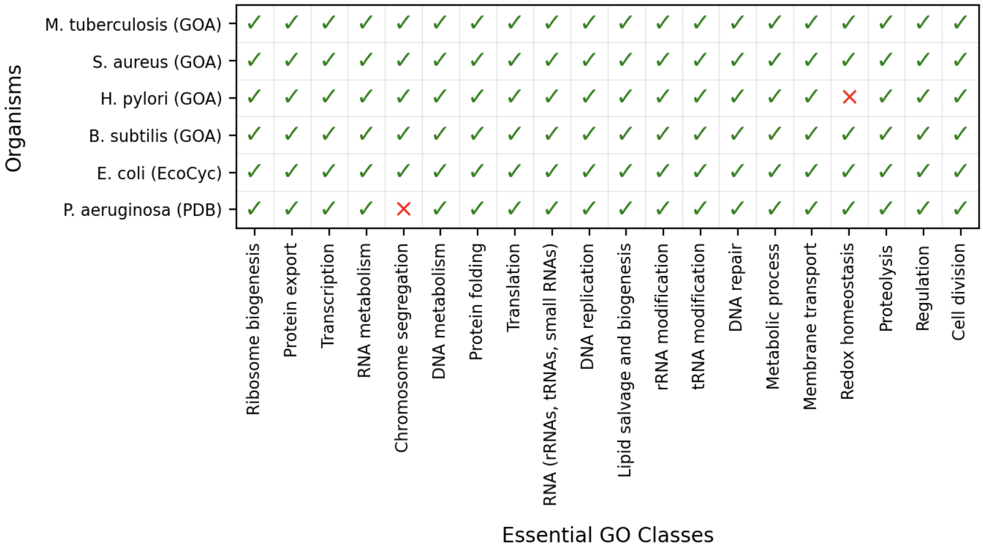

Fig. S1. Presence or absence of 'Core' GO classes in genome annotations from model organisms, with checked cells indicating presence of at least one annotation to the corresponding class.

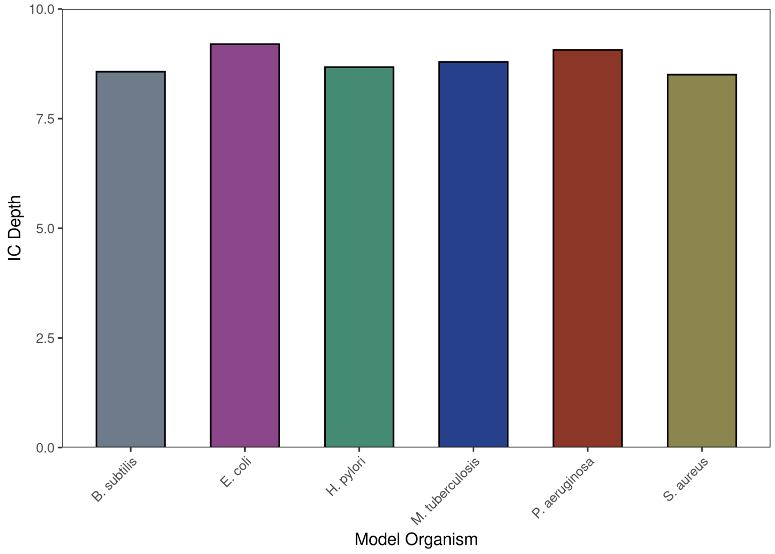

Fig. S2. Information Content (IC) depth for specific GO class annotations across six bacterial model organisms: *E. coli*, *B. subtilis*, *P. aeruginosa*, *H. pylori*, *S. aureus*, and *M. tuberculosis*.

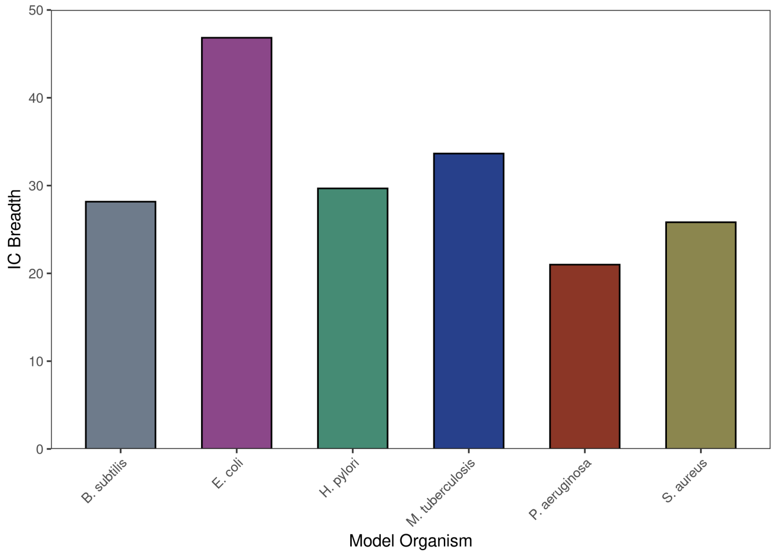

Fig. S3. Information Content (IC) breadth normalized by the number of proteins for specific GO class annotations across six bacterial model organisms: *E. coli*, *B. subtilis*, *P. aeruginosa*, *H. pylori*, *S. aureus*, and *M. tuberculosis*.

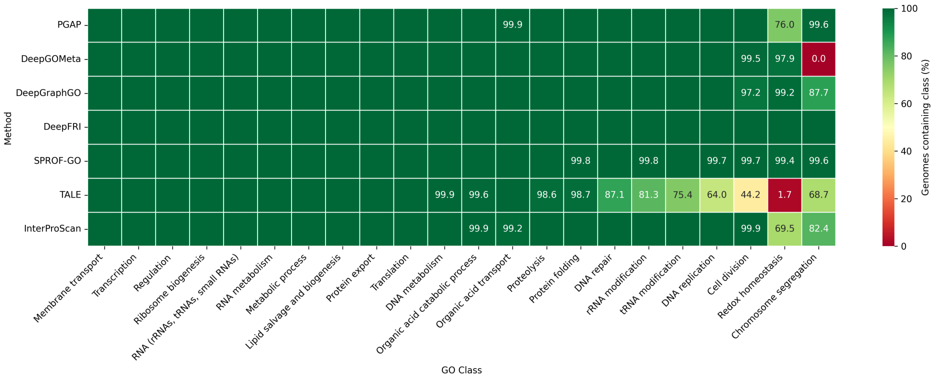

Fig. S4. Evaluation of essential GO class presence in bacterial genomes annotated using several methods. Numeric labels highlight <100% presence, while a value of 100% is indicated by the solid green color without text for visual clarity.

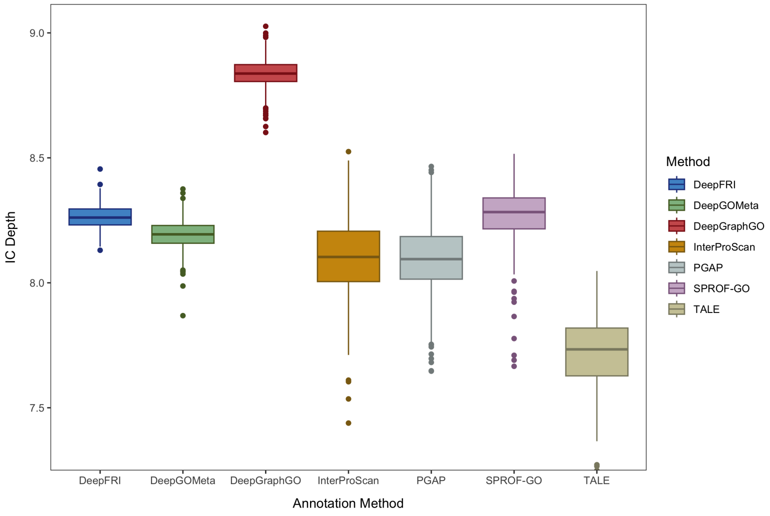

Fig. S5. Distribution of Information Content (IC) depth for specific GO classes across the seven methods.

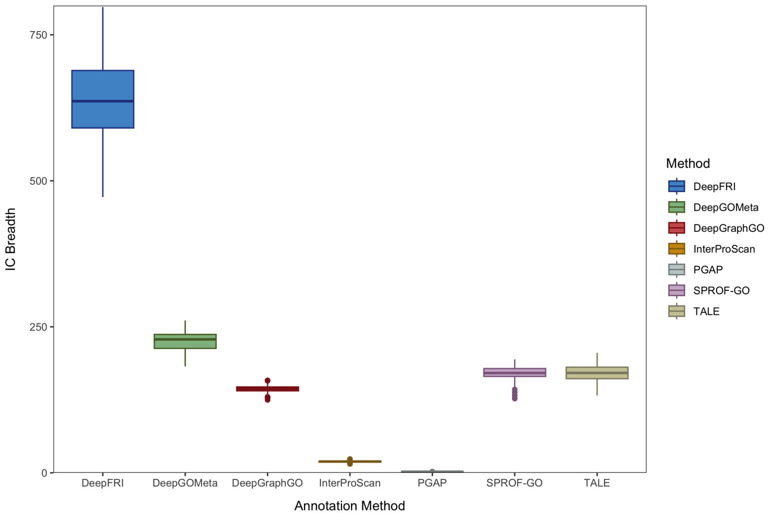

Fig. S6. Distribution of Information Content (IC) breadth for specific GO classes across the seven methods.

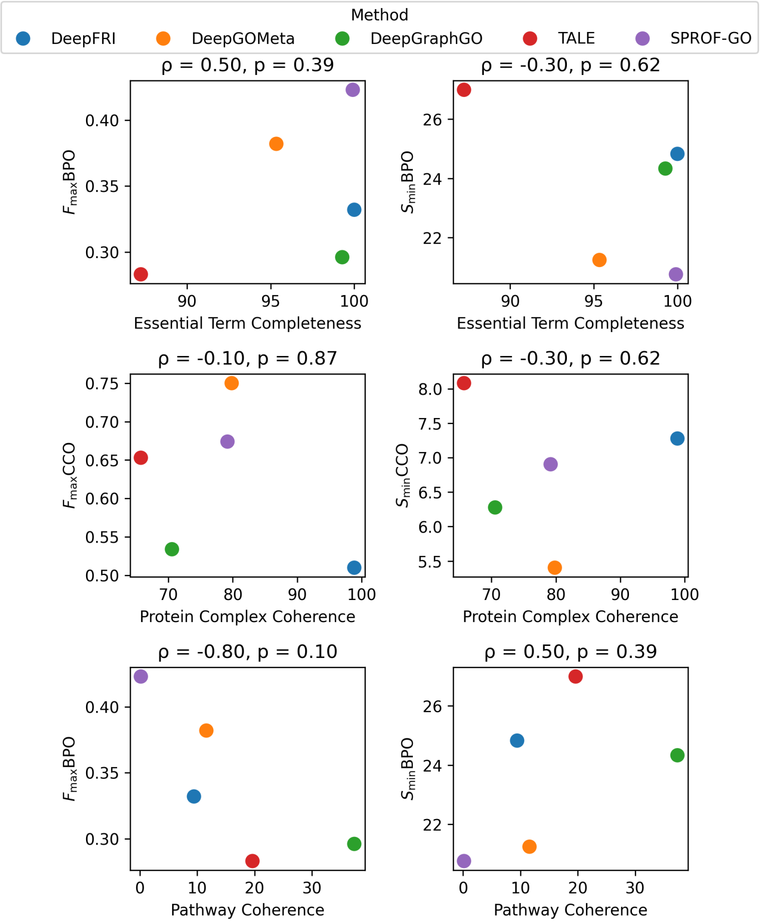

Fig. S7. Spearman correlations between evaluation metrics ($F_{\max}$, $S_{\min}$) and framework evaluation metrics (Essential term completeness, protein complex coherence, and pathway coherence).

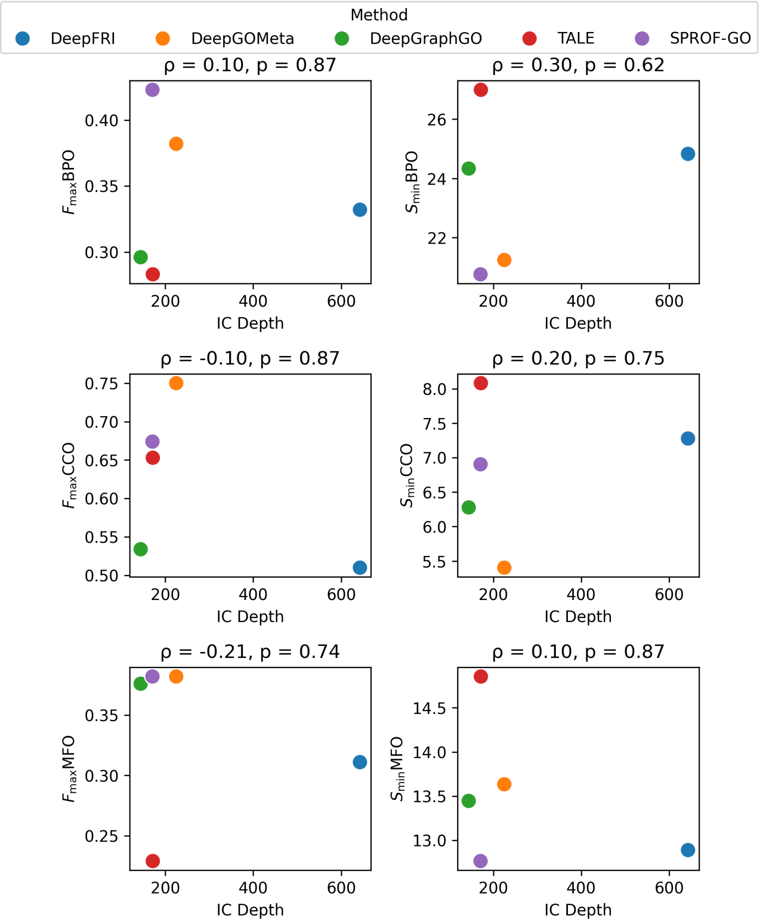

Fig. S8. Spearman correlations between evaluation metrics ($F_{\max}$, $S_{\min}$) and Information Content (IC) depth for all GO subdomains (BPO, CCO, and MFO) based on annotation method results.

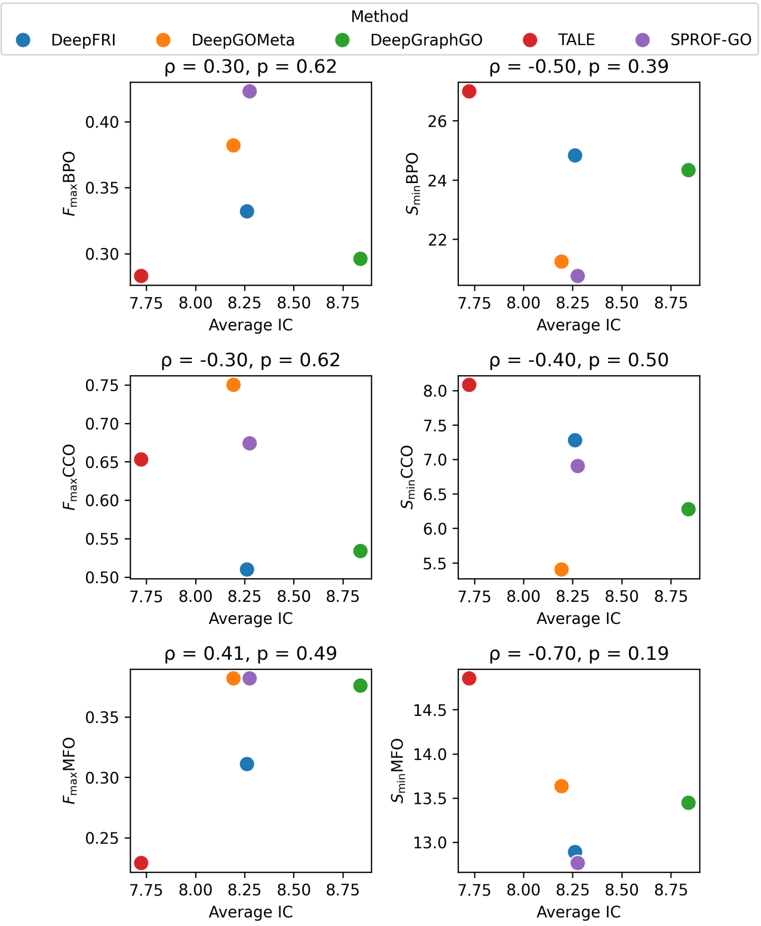

Fig. S9. Spearman correlations between evaluation metrics ($F_{\max}$, $S_{\min}$) and average Information Content (IC) for all GO subdomains (BPO, CCO, and MFO) based on annotation method results.

3. Tables

Method	MFO	CCO	BPO
SPROF-GO	0.13	0.54	0.13
DeepGOMeta	0.27	0.27	0.11
TALE	0.28	0.56	0.15
DeepFRI	0.28	0.01	0.01
DeepGraphGO	0.33	0.21	0.30

Table S1. Threshold that maximized $F_{\max}$ for each Gene Ontology (GO) sub-ontology for each method.

Category	Function	GO Class Name	GO Class
Core	DNA metabolism	DNA metabolic process	GO:0006259
	DNA replication	DNA replication	GO:0006260
	DNA repair	DNA repair	GO:0006281
	Transcription	DNA-templated transcription	GO:0006351
	Translation	Translation	GO:0006412
	Cell division	Cell division	GO:0051301
	Chromosome segregation	Chromosome segregation	GO:0007059
	Ribosome biogenesis	Ribosome biogenesis	GO:0042254
	Protein folding	Protein folding	GO:0006457
	Protein export	Protein transport	GO:0015031
	RNA metabolism	RNA metabolic process	GO:0016070
	rRNA modification	rRNA modification	GO:0000154
	tRNA modification	tRNA modification	GO:0006400
	RNA (rRNAs, tRNAs, small RNAs)	RNA biosynthetic process	GO:0032774
	Proteolysis	Proteolysis	GO:0006508
	Metabolic processes	Metabolic process	GO:0008152
	Membrane transport	Transmembrane transport	GO:0055085
	Lipid salvage and biogenesis	Lipid metabolic process	GO:0006629
	Transport of nonglucose carbon sources	Organic acid transport	GO:0015849
	Catabolism of nonglucose carbon sources	Organic acid catabolic process	GO:0016054
	Redox homeostasis	Cell redox homeostasis	GO:0045454
	Regulation	Regulation of biological process	GO:0050789
Glucose Metabolism	Glycolysis	Glycolytic process	GO:0006096
	Glucose transport	Glucose transmembrane transport	GO:1904659
Environmental Adaptation	Mobile elements	Transposition	GO:0032196
	DNA topology	DNA conformation change	GO:0071103
	Lipoprotein	Lipoprotein metabolic process	GO:0042157
Nutrient Uptake	Cofactor transport and salvage	Vitamin transmembrane transporter activity	GO:0090482
	Acylglycerol breakdown	Acylglycerol catabolic process	GO:0046464
	Nucleotide salvage	Nucleotide salvage	GO:0043173
Defense	DNA restriction	DNA restriction-modification system	GO:0009307
	Efflux	Export across plasma membrane	GO:0140115

Table S2. Functional categories from syn1.0 manually mapped to the most relevant Gene Ontology (GO) class.

Model Organism	Processes	Pathways	Complexes
B. subtilis	94.00%	61.84%	63.51%
E. coli	96.25%	82.84%	84.23%
H. pylori	92.77%	45.10%	73.21%
M. tuberculosis	95.98%	61.45%	72.97%
P. aeruginosa	83.90%	20.83%	72.73%
S. aureus	93.29%	48.39%	65.67%

Table S3. Percentage of coherent processes, MetaCyc pathways, and protein complexes across selected model organisms.

References

1. Wyatt T. Clark and Predrag Radivojac. Information-theoretic evaluation of predicted ontological annotations. *Bioinformatics*, 29(13):i53–i61, June 2013.
2. Rund Tawfiq, Kexin Niu, et al. Deepgometa for functional insights into microbial communities using deep learning-based protein function prediction. *Scientific Reports*, 14(1), December 2024.
3. Clyde A Hutchison III, Ray-Yuan Chuang, Vladimir N Noskov, Nacyra Assad-Garcia, Thomas J Deerinck, Mark H Ellisman, John Gill, Krishna Kannan, Bogumil J Karas, Li Ma, et al. Design and synthesis of a minimal bacterial genome. *Science*, 351(6280):aad6253, 2016.
